# Pain in People Living With Obesity: Baseline Multidimensional Profiles, Prevalence and Biopsychosocial Factors From a Cohort Study

**DOI:** 10.1002/ejp.70317

**Published:** 2026-07-03

**Authors:** Natasha S. Hinwood, Colin G. Dunlevy, Catherine Doody, Catherine Blake, Bróna M. Fullen, Gráinne O′Donoghue, Jean O′Connell, Carel W. Le Roux, Francis M. Finucane, Susie Birney, Fionnuala Fildes, Keith M. Smart

**Affiliations:** ^1^ UCD School of Public Health, Physiotherapy and Sport Science University College Dublin Dublin Ireland; ^2^ UCD Centre for Translational Pain Research University College Dublin Dublin Ireland; ^3^ Physiotherapy Department The Beacon Hospital Dublin Ireland; ^4^ Centre for Obesity Management (COM) St Columcille's and St Vincent's University Hospitals Dublin Ireland; ^5^ UCD School of Medicine University College Dublin Dublin Ireland; ^6^ Diabetes Complications Research Centre University College Dublin Dublin Ireland; ^7^ School of Medicine, College of Nursing and Health Sciences University of Galway Galway Ireland; ^8^ Bariatric Medicine Service, Centre for Diabetes, Endocrinology and Metabolism Galway University Hospitals Galway Ireland; ^9^ Irish Coalition for People Living With Obesity (ICPO) Dublin Ireland; ^10^ St. Vincent's Private Hospital Dublin Ireland

**Keywords:** BMI, longitudinal cohort study, nociplastic, obesity, pain profiling

## Abstract

**Background:**

Obesity is considered a risk factor for pain, and comorbid obesity and pain have a cumulatively worse impact on function and quality of life than either condition alone. The aim of this study was to estimate the prevalence of pain and describe the multidimensional biopsychosocial pain profiles of people with obesity (PwO).

**Methods:**

This pre‐specified cross‐sectional study reports the baseline data from a longitudinal cohort study. We recruited 519 PwO from three specialist obesity clinics in Ireland. Participants completed pain‐, obesity‐ and health‐related questionnaires to capture the multidimensional biopsychosocial characteristics of their pain experience. Data were analysed using descriptive and inferential statistics.

**Results:**

Pain prevalence was 77% (95% CI: 73.1%–80.6%) (70.7% female; mean age 46.6 ± 12.7 years). Participants' pain characteristics reflected heterogeneity in the pain experiences of PwO, including (mean; SD): pain intensity (0–10 numerical rating scale) (3.97 ± 2.9), number of pain locations (0–35) (5.06 ± 5.3), levels of pain‐related disability and self‐efficacy. The prevalence of nociplastic pain was 54% (95% CI: 49.3%–58.6%) and neuropathic pain was 30% (95% CI: 25.6%–34.8%). Clinically significant levels of pain‐related worrying and kinesiophobia were reported by 20.9% (95% CI: 17.3%–24.8%) and 49.9% (95% CI: 45%–54.7%) of participants.

**Conclusion:**

The majority (77%) of PwO attending specialist obesity treatment services report experiencing pain. The intensity, nature, and impact of their pain vary. Over half reported nociplastic pain, one‐third neuropathic pain, one‐fifth significant pain‐related worrying, and half kinesiophobia. These findings have implications for pain management in PwO.

**Significance Statement:**

This is the first multicentre prospective cohort study to investigate the multidimensional pain profiles of PwO. Pain prevalence was 77%. This is the first study to estimate (i) baseline prevalence of nociplastic‐dominant pain in PwO (54%); (ii) baseline prevalence of neuropathic pain in PwO (30%); (iii) clinically significant levels of pain‐related fear (20.9%); and (iv) clinically significant levels of kinesiophobia (49.9%), in PwO attending specialist obesity treatment services. These findings have clinical implications for the treatment of pain in PwO.

**Abbreviations**
BMI: body mass index
CP: chronic Pain
CPAP: continuous positive airway pressure
CSI: Central Sensitisation Inventory
EOSS: Edmonton Obesity Staging System
EQ‐5D‐5L: EuroQol‐ 5 dimension
KOSC: King's Obesity Staging Criteria
LEFS: Lower Extremity Functional Scale
MBM: Michigan Body Map
NPQ: Neuropathic Pain Questionnaire
NRS: Numeric Rating Scale
NSAIDs: non‐steroidal anti‐inflammatories
PCS: Pain Catastrophisation Scale
PDI: Pain Disability Index
PHQ9: Patient Health Questionnaire
PMP: Pain Management Programme
PPI: Public Patient Involvement
PROMs: Patient Reported Outcome Measures
PSEQ: Pain Self‐efficacy Scale
PwO: People/Person living with Obesity
RMDQ: Roland Morris Disability Questionnaire
SCQ: Self‐Administered Comorbidity Questionnaire
TSK‐11: Tampa Scale of Kinesiophobia
UEFI‐15: Upper Extremity Functional Index
WEMWBS: Warwick‐Edinburgh Mental Well‐Being Scale

## 1 Introduction

The disease burdens of both pain and obesity have been well‐documented (Chin et al. 2020; Breen et al. 2022; Pickering et al. 2025; Rubino et al. 2025). Prevalence estimates of pain in people living with obesity (PwO) vary greatly, ranging from 17.9% (Zhong et al. 2024) to 91% (MacLellan et al. 2017) likely due to differences in case definitions and cohort settings. Estimates vary depending on associated pain mechanisms (nociceptive, neuropathic, nociplastic) and related pathologies (e.g., rheumatological conditions, diabetes, low back pain) (Chin et al. 2020; Garcia et al. 2024; Narouze and Souzdalnitski 2015). Obesity is both a risk factor for developing pain (Zhou et al. 2024) and is associated with increased pain intensity, interference, pain‐related disability, socioeconomic distress and poorer psychological well‐being compared to people not living with obesity (Narouze and Souzdalnitski 2015; Garcia et al. 2024; Vos et al. 2020). Furthermore, obesity is common among those living with chronic pain (Chin et al. 2020; Okifuji and Hare 2015). Cohort studies and systematic reviews have consistently reported a strong association between adiposity (as measured through body mass index (BMI)) and an increased prevalence of musculoskeletal pain (Chin et al. 2020; Narouze and Souzdalnitski 2015; Shiri et al. 2010; Stone and Broderick 2012).

Evidence suggests that pain in PwO may be more complex compared to those without obesity, including (i) more widespread (Chin et al. 2020; Narouze and Souzdalnitski 2015), (ii) leading to altered pain perception and (iii) be barrier to weight‐loss and more challenging to manage (Garcia et al. 2024; Malfliet et al. 2021). Limited evidence from both clinical and preclinical studies suggests that integrated strategies targeting both obesity and chronic pain management lead to improvements in pain and disability compared with either intervention alone (Dunlevy et al. 2019; Pudalov et al. 2021; Ryan et al. 2017). However, barriers to pain management and care for PwO persist (Norman et al. 2022).

Mechanisms linking pain and obesity are complex, and may include mechanical loading, inflammation, and psychological status (Garcia et al. 2024; Narouze and Souzdalnitski 2015). However, the degree and nature of how mechanisms of pain may differ between people with and without obesity remain unclear. The biopsychosocial model of pain recognises the biological (physiology, genetics), psychological (thoughts, beliefs, behaviours, emotions) and social (relationships, sociocultural influences) determinants of people's pain experiences and is widely endorsed in contemporary clinical practice (Cohen et al. 2021; Smart 2023; Sullivan and de C Williams 2025). Understanding how these multidimensional, bidirectional and dynamic factors interact is fundamental to providing efficient and effective care and lessening disease burden. The biopsychosocial multidimensional characteristics of pain in PwO remain underexplored. The aim of this study was to investigate the biopsychosocial multidimensional pain experiences of PwO attending obesity treatment services, including its prevalence, severity, location and potential underlying mechanisms, as well as pain‐related disability, social function and emotional wellbeing (Smart et al. 2022).

## 2 Aims

The primary aims of this study were to estimate the point prevalence of pain and characterise the multidimensional biopsychosocial pain profiles of people living with obesity attending obesity treatment services.

## 3 Methods

Detailed study methods have been reported in a study protocol (Smart et al. 2022) and are summarised below.

### 3.1 Design

This study presents the baseline cross‐sectional pain profiles of participants recruited to a multicentre longitudinal cohort study, registered on the Open Science Framework in January 2022 (DOI: https://doi.org/10.17605/OSF.IO/QCWUE). These cross‐sectional results represent the baseline results of the subsequent longitudinal study. We report our findings in accordance with the Strengthening the Reporting of Observational Studies in Epidemiology (STROBE) (von Elm 2008) and Guidance for Reporting Involvement of Patients and the Public (GRIPP 2) (Staniszewska et al. 2017) guidelines (Supporting Information S1 and S2).

### 3.2 Data Collection

Participants included in this study were adults aged ≥ 18 years and newly attending an obesity treatment service, while participants unable to consent were excluded from participation. Participants were recruited using a convenience sampling approach based on monthly clinic intake, aiming to recruit the largest sample feasible. A priori calculations confirmed the adequacy of the sample size for cross‐sectional prevalence estimates (*n* = 196). Baseline demographic, anthropometric, pain and general health data were collected in‐person or remotely from consenting patients via paper or online questionnaires (accessed via email, SMS or QR code). Participants were invited to complete 15 pain and health‐related patient‐reported outcome measures (PROMs) reflecting the multiple biopsychosocial dimensions of the pain experience. These included pain intensity (Numeric Rating Scale [NRS], range 0–10) (Jensen and Karoly 2011); pain location (Michigan Body Map [MBM], range 0–35) (Brummett et al. 2016), pain‐related disability (i. Upper Extremity Functional Index [UEFI‐15], range 0–100 (Chesworth et al. 2014); ii. Lower Extremity Functional Scale [LEFS], range 0–80 (Binkley et al. 1999); iii. Roland Morris Disability Questionnaire [RMDQ], range 0–24) (Macedo et al. 2011), social function (Pain Disability Index [PDI], range 0–70) (Pollard 1984), pain mechanisms (i. Central Sensitization Index [CSI], range 0–100) (Neblett et al. 2013); (ii. Neuropathic Pain Questionnaire [NPQ]) (Krause and Backonja 2003), pain‐related worrying (Pain Catastrophizing Scale [PCS], range 0–52) (Sullivan et al. 1995), pain self‐efficacy (Pain Self‐Efficacy Questionnaire [PSEQ], range 0–60) (Nicholas 2007), kinesiophobia (Tampa Scale of Kinesiophobia [TSK‐11], range 11–48) (Woby et al. 2005), comorbidities (Self‐reported Comorbidities Questionnaire [SCQ], range 0–36) (Sangha et al. 2003), health‐related quality of life (EuroQol‐5 Dimension [EQ‐5D‐5L]) (Feng et al. 2021), and emotional wellbeing (i. Patient Heath Questionnaire [PHQ9] range 0–27) (Cameron et al. 2008), (Warwick Edinburgh Mental Well‐Being Scale [WEMWBS], range 14–70) (Tennant et al. 2007). Questionnaires were checked for completeness and participants were encouraged to complete any missing questions, to reduce missing data for questionnaires collected in‐person or electronically. Missing demographic/anthropometric data (e.g., baseline age, weight or height data) were collected from patient records where possible.

Attrition bias was limited using both follow‐up reminder strategies and barrier‐reduction strategies (providing pre‐paid return‐envelopes for paper questionnaires, and where participants expressed difficulties with reading or writing, a4 researcher (NH) administered the questionnaire in a standardised verbal format) (Teague et al. 2018). Baseline data collection commenced in January 2022 and was completed by October 2023.

### 3.3 Statistical Analysis

Data were cleaned and prepared for analysis using Statistical Package for the Social Sciences (SPSS) (v. 29). Data were summarised using descriptive statistics and point prevalence estimates were calculated with 95% confidence intervals. Data were assessed for normality visually and statistically (Shapiro–Wilk), with exploratory parametric testing applied in consultation with the study biostatistician. Exploratory analyses tested for between‐group differences in pain‐related PROMs according to gender and BMI classification (Class I (30–34.9 kg/m^2^); II (35–39.9 kg/m^2^); and III (≥ 40 kg/m^2^)), (*t*‐tests, Chi‐Squared and One‐Way ANOVA), and pairwise effect sizes were estimated using Cohen's *d* (*d*).

Where data were missing, we undertook an available case analysis for each outcome variable. Patterns of missing data were discussed with members of the research team and agreed to be most likely missing at random for all outcome measures, except for the SCQ. The proportion of missing data ranged from 1.7% (NRS, RMDQ) to 35.8% (SCQ). We speculate that the proportion of missing data for the SCQ was due to the length and design (a complex branching logic) of this outcome measure.

### 3.4 Deviations From Protocol

We took the following decisions post hoc to the publication of the protocol but prior to undertaking data analyses:

- In the absence of a universally accepted definition for ‘multisite pain’, we operationally define the presence of multisite pain based on a score of ≥ 3 pain sites on the MBM (MacLellan et al. 2017);
- Baseline prevalence estimates of pain rumination (repeated worry), pain magnification (evaluation of the pain as a threat), and pain helplessness (belief that nothing can help to resolve the pain) (Wheeler et al. 2019) were estimated using sub‐scales of the PCS, which have been previously described (Cresswell et al. 2020);
- The concept of ‘*pain catastrophizing*’ has been described in this study as ‘*pain‐related worrying*’, consistent with current evidence supporting the use of patient‐centred language (Crombez et al. 2020);
- The prevalence of clinically significant kinesiophobia was inferred from scores of ≥ 25 on the TSK‐11 (Roelofs et al. 2004; Woby et al. 2005);
- Data from participants collected using the SCQ were trichotomized based on participants reporting zero, one, or ≥ 2 comorbidities consistent with other studies (Ochsenkuehn et al. 2022; Sangha et al. 2003).

## 4 Results

**FIGURE 1:**
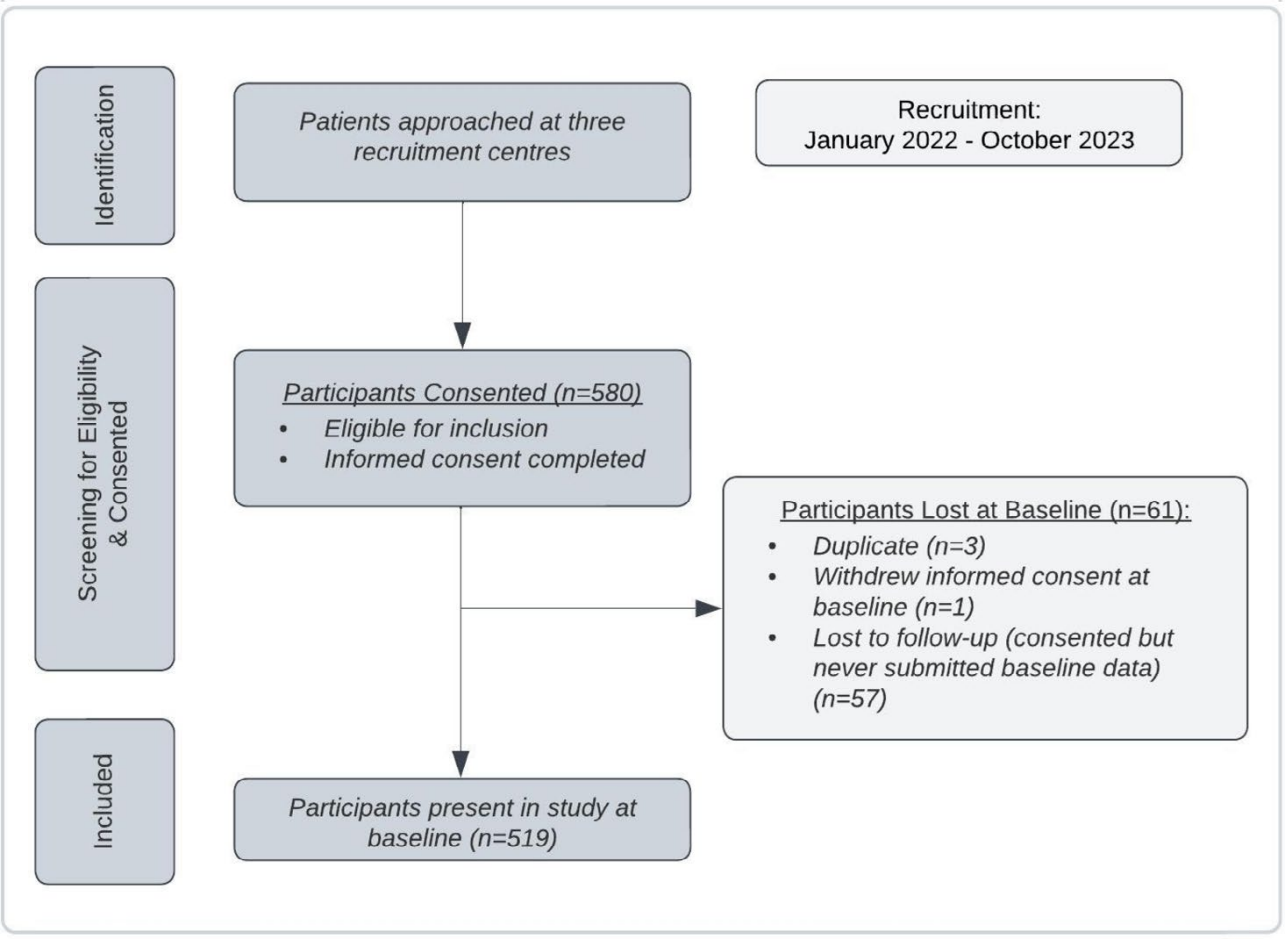
Patient flow chart.

Of the 580 participants who initially consented to participate, 519 entered the study, of whom 57% and 43% were recruited through publicly (Health Service Executive) and privately funded clinics, respectively. Figure 1 illustrates participant enrolment into the study at the cross‐sectional baseline. Demographic, anthropometric, PROMs and missing data proportions are presented in Tables 1A and 1B. Data are reported for the total cohort unless where indicated.

**TABLE 1A:** Baseline results for all sociodemographic and health‐related PROMs.

		Total sample					Female				Male			
		*n* =^a^	Mean	SD	Range	Missing *n* (%)	*n* =	Mean	SD	Range	*n* =	Mean	SD	Range
Sociodemographic														
Age		519	46.61	12.65	18–76	0	367	46.42	12.62	19–76	147	47.73	12.43	18–76
Gender	Total	519				0								
	Female	367 (70.7%)												
	Male	147 (28.3%)												
	Prefer not to say	2 (0.4%)												
	Other	3 (0.6%)												
Ethnic background	Total	519				0	367				147			
	White	499 (96.1%)					356 (97%)				139 (94.6%)			
	Black	5 (1%)					4 (1.1%)				1 (0.7%)			
	Asian	8 (1.5%)					4 (1.1%)				4 (2.7%)			
	Other	7 (1.3%)					3 (0.8%)				3 (2%)			
Relationship status	Total	499				20 (3.9%)	353				141			
	Single	126 (25.3%)					85 (24.1%)				39 (27.7%)			
	Married	275 (55.1%)					200 (56.7%)				74 (52.5%)			
	Partnered	52 (10.4%)					35 (9.9%)				16 (11.3%)			
	Separated	16 (3.2%)					12 (3.4%)				4 (2.8%)			
	Divorced	8 (1.6%)					7 (2%)				1 (0.7%)			
	Widowed	11 (2.2%)					5 (1.4%)				6 (4.3%)			
	Prefer not to say	11 (2.2%)					9 (2.5%)				1 (0.7%)			
Employment status	Total	519				0	367				147			
	Working Full‐time	253 (48.7%)					163 (44.4%)				90 (61.2%)			
	Working Part‐time	61 (11.8%)					53 (14.4%)				7 (4.8%)			
	Retired	44 (8.5%)					34 (9.3%)				10 (6.8%)			
	Unable to work due to disability	96 (18.5%)					64 (17.4%)				29 (19.7%)			
	Homemaker	32 (6.2%)					32 (8.7%)				0			
	Student	9 (1.7%)					6 (1.6%)				2 (1.4%)			
	Unemployed	24 (4.6%)					15 (4.1%)				9 (6.1%)			
Education background	Total	518				1 (0.2%)	366				147			
	No formal education	4 (0.8%)					3 (0.8%)				1 (0.7%)			
	Primary education	23 (4.4%)					19 (5.2%)				4 (2.7%)			
	Lower secondary education	54 (10.4%)					34 (9.3%)				19 (12.9%)			
	Higher secondary education	77 (14.9%)					53 (14.5%)				23 (15.6%)			
	Technical or vocational qualification	68 (13.1%)					51 (13.9%)				15 (10.2%)			
	Third level (non‐degree)	96 (18.5%)					65 (17.8%)				31 (21.1%)			
	Third level (degree)	116 (22.4%)					84 (23%)				31 (21.1%)			
	Postgraduate degree	80 (15.4%)					57 (15.6%)				23 (15.6%)			
Obesity and health‐related outcomes														
Smoking status	Total	515				4 (0.8%)	364				146			
	Yes, daily	42 (8.2%)					32 (8.8%)				9 (6.2%)			
	Yes, at least once a week	6 (1.2%)					5 (1.4%)				1 (0.7%)			
	Yes, less often than once a week	14 (2.7%)					6 (1.6%)				8 (5.5%)			
	No, not at all	453 (88%)	95% CI: 84.8%–90.6%				321 (88.2%)				128 (87.7%)			
BMI (kg/m^2^)	Total	516	46.81	9.14	26–105.5	3 (0.6%)	365	46.63	8.09	26–84.8	146	47.15	11.32	30.2–105.5
	BMI 25–29.9 kg/m^2^	2 (0.4%)					2 (0.5%)				0			
	Class I (BMI 30–34.9 kg/m^2^)	27 (5.2%)					18 (4.9%)				9 (6.2%)			
	Class II (BMI 35–39.9 kg/m^2^)	71 (13.8%)					42 (11.5%)				27 (18.5%)			
	Class III (BMI ≥ 40 kg/m^2^)	416 (80.6%)					303 (83%)				110 (75.3%)			

King's obesity staging criteria (KOSC)		*n* = 44					*n* = 30				*n* = 14			
	Stage	0 Normal health	1 At risk	2 Established disease	3 Advanced disease		0 Normal health	1 At risk	2 Established disease	3 Advanced disease	0 Normal health	1 At risk	2 Established disease	3 Advanced disease
	Airway	7 (15.9%)	23 (52.3%)	13 (29.5%)	1 (2.3%)		5 (16.7%)	20 (66.7%)	5 (16.7%)	0	2 (14.3%)	3 (21.4%)	8 (57.1%)	1 (7.1%)
	BMI	2 (4.5%)	2 (4.5%)	36 (81.8%)	4 (9.1%)		2 (6.7%)	2 (6.7%)	25 (83.3%)	1 (3.3%)	0	0	11 (78.6%)	3 (21.4%)
	Cardiovascular	14 (31.8%)	19 (43.2%)	9 (20.5%)	2 (4.5%)		11 (36.7%)	1 (40%)	2 (23.3%)	0	3 (21.4%)	7 (50%)	2 (14.3%)	2 (14.3%)
	Diabetes	29 (65.9%)	4 (9.1%)	10 (22.7%)	1 (2.3%)		21 (70%)	3 (10%)	6 (20%)	0	8 (57.1%)	1 (7.1%)	4 (28.6%)	1 (7.1%)
	Economic	24 (54.5%)	14 (31.8%)	6 (13.6%)	0		16 (53.3%)	10 (33.3%)	4 (13.3%)	0	8 (57.1%)	4 (28.6%)	2 (14.3%)	0
	Functional	4 (9.1%)	35 (75.9%)	5 (11.4%)	0		2 (6.7%)	25 (83.3%)	3 (10%)	0	2 (14.3%)	10 (71.4%)	2 (14.3%)	0
	Gonadal Axis	29 (65.9%)	9 (20.5%)	6 (13.6%)	0		20 (66.7%)	7 (23.3%)	3 (10%)	0	9 (64.3%)	1 (14.3%)	3 (21.4%)	0
	Health Status (Perceived)	5 (11.4%)	33 (75%)	5 (11.4%)	1 (2.3%)		1 (3.3%)	26 (86.7%)	3 (10%)	0	4 (28.6%)	7 (50%)	2 (14.3%)	1 (7.1%)
	Image	5 (11.4%)	31 (70.5%)	7 (15.9%)	1 (2.3%)		3 (10%)	20 (66.7%)	7 (23.3%)	0	2 (14.3%)	11 (78.6%)	0	1 (7.1%)
	Junction of gastro‐oesophagus	23 (52.3%)	13 (29.5%)	8 (18.2%)	0		14 (46.7%)	9 (30%)	7 (23.3%)	0	9 (64.3%)	4 (28.6%)	1 (7.1%)	0
	Kidney	31 (70.5%)	2 (4.5%)	11 (25%)	0		19 (63.3%)	0	11 (36.7%)	0	12 (85.7%)	2 (14.3%)	0	0
	Liver	34 (77.3%)	5 (11.4%)	5 (11.4%)	0		23 (76.7%)	5 (16.7%)	2 (6.7%)	0	11 (78.6%)	0	3 (21.4%)	0
	Medication	35 (79.5%)	8 (18.2%)	1 (2.3%)	0		25 (83.3%)	5 (16.7%)	0	0	10 (71.4%)	3 (21.4%)	1 (7.1%)	0
	Neurology	44 (100%)	0	0.00	0		30 (100%)	0	0	0	14 (100%)	0	0	0
	Other	44 (100%)	0	0.00	0		29 (96.7%)	0	1 (3.3%)	0	14 (100%)	0	0	0
Edmonton obesity staging system (EOSS)		*n* = 79					*n* = 55				*n* = 23			
	Stage 1	0					0				0			
	Stage 2	27 (34.2%)					22 (40%)				5 (21.7%)			
	Stage 3	49 (62%)					33 (60%)				15 (65.2%)			
	Stage 4	3 (3.8%)					0				3 (13%)			
Comorbidities	Self‐Administered Comorbidity Questionnaire (SCQ) (0–36)	333	5.82	4.41	0–22	186 (35.8%)	237	5.77	4.31	0–22	93	5.89	4.68	0–20
	Nil comorbidities	37 (11.1%)					28 (11.8%)				9 (9.7%)			
	1 comorbidity	62 (18.6%)					39 (16.5%)				22 (23.7%)			
	≥ 2 comorbidities	234 (70.3%)					170 (71.7%)				62 (66.7%)			
Health‐related	EuroQol‐ 5 dimension (EQ‐5D‐5L): Utility Score (< 0 − +1)	498	0.57	0.39	−0.78–1	21 (4%)	352	0.55	0.02	−0.78–1	142	0.61	0.35	−0.78–1
Quality of life	EQ‐5D‐5L: VAS Score (0–100)	500	54.22	20.30	0–100	19 (3.7%)	352	54.21	20.66	0–100	144	54.17	19.56	0–100
Depression	Patient Health Questionnaire (PHQ9) (0–27)	480	10	6.99	0–27	39 (7.5%)	337	10.48	7.061	0–27	139	8.69	6.57	0–27
	No/minimal depression (0–4)	120 (25%)					77 (22.8%)				42 (30.2%)			
	Mild depression (5–9)	131 (27.3%)					88 (26.1%)				43 (30.9%)			
	Moderate depression (10–14)	107 (22.3%)					78 (23.1%)				28 (20.1%)			
	Moderately severe depression (15–19)	65 (13.5%)					49 (14.5%)				15 (10.8%)			
	Severe depression (20–27)	57 (11.9%)					45 (13.4%)				11 (7.9%)			
Mental	Warwick‐Edinburgh	480	44.47	11.62	14–70	39 (7.5%)	340	43.89	11.76	14–70	135	46.15	11.28	14–70
	Mental well‐being													
Well‐being	Scale (WEMWBS) (14–70)													

*Note:* Participants who reported their gender as ‘*other*’ (*n* = 3) or ‘preferred not to say’ (*n* = 2) were excluded from stratified analysis based on gender due to insufficient sample size for meaningful comparison. Confidence Intervals (95% CI) calculated (binomial) to 95% on 15.12.24: https://sample‐size.net/confidence‐interval‐proportion/.

Abbreviations: BMI, Body Mass Index (Kg/m^2^); n, number of respondents; PROMs, Patient Reported Outcome Measures; SD, Standard Deviation.

^a^ *n* = Valid Sample.

**TABLE 1B:** Baseline results for all pain‐related PROMs.

		Total sample					Female				Male			
		*n* =^a^	Mean	SD	Range	Missing *n* (%)	*n* =	Mean	SD	Range	*n* =	Mean	SD	Range
Pain outcomes and PROMs														
Pain presence	Total	508				11 (2.1%)	361				143			
	I have no pain	117 (23%)					76 (21.1%)				41 (28.7%)			
	I have pain, but am receiving treatment	163 (32.1%)					128 (35.5%)				33 (23.1%)			
	I have pain, but am not receiving treatment	228 (44.9%)					157 (43.5%)				69 (48.3%)			
	Pain Prevalence	391 (77%)	95% CI: 73.1%–80.6%				285 (78.9%)	95% CI: 74.4%—83%			102 (71.3%)	95% CI: 63.2%—78.6%		
Pain intensity	Numeric Rating Scale (NRS) (0–10)	510	3.97	2.91	0–10	9 (1.7%)	364	4.17	2.97	0–10	141	3.43	2.73	0–10
	No Pain	105 (20.6%)					74 (20.3%)				31 (22%)			
	Mild Pain (1–4)	171 (33.5%)					108 (29.7%)				61 (43.3%)			
	Moderate Pain (5–6)	122 (23.9%)					93 (25.5%)				27 (19.1%)			
	Severe Pain (≥ 7)	112 (22%)					89 (24.5%)				22 (15.6%)			
Pain location	Michigan Body Map (MBM) (0–35)	519	5.06	5.36	0–35	0	366	5.54	5.64	0–35	147	3.53	3.59	0–16
	No pain sites	117 (22.6%)					75 (20.5%)				42 (28.6%)			
	1 Pain Site	34 (6.6%)					22 (6%)				12 (8.2%)			
	2 Pain Sites	49 (9.4%)					37 (10.1%)				11 (7.5%)			
	Prevalence Multisite pain (≥ 3 sites)	319 (61.4%)	95% CI: 57.1%–65.6%				232 (63.4%)	95% CI: 58.2%–68.3%			82 (55.8%)	95% CI: 47.4%–64%		
Pain‐related disabiliTY	Upper Extremity Functional Index UEFI‐15 (Interval score: 0–100)	458	79.09	23.76	0–100	61 (11.8%)	323	77.11	25.06	0–100	132	86.24	18.83	0–100
	Lower Extremity Functional Scale (LEFS) (0–80)	475	47.44	21.75	0–80	44 (8.5%)	338	46.13	21.9	2–80	132	50.9	21.15	0–80
	Roland Morris Disability Questionnaire (RMDQ) (0–24)	510	5.56	6.31	0–24	9 (1.7%)	360	5.82	6.42	0–24	145	4.81	5.96	0–22
Social impact of pain	Pain Disability Index (PDI) (0–70)	463	24.83	19.60	0–70	56 (10.8%)	328	24.84	19.77	0–70	132	24.67	19.19	0–70
Central sensitisation	Central Sensitisation Inventory (CSI) (0–100)	454	42.08	17.62	0–100	65 (12.5%)	322	44.11	17.34	0–100	127	36.12	16.75	0–78
	Subclinical (0–29)	103 (22.7%)					63 (19.6%)				40 (31.5%)			
	Mild (30–39)	106 (23.3%)					68 (21.1%)				37 (29.1%)			
	Moderate (40–49)	90 (19.8%)					65 (20.2%)				25 (19.7%)			
	Severe (50–59)	79 (17.4%)					64 (19.9%)				15 (11.8%)			
	Extreme (60–100)	76 (16.7%)					62 (19.3%)				10 (7.9%)			
	Prevalence (CSI ≥ 40)	245 (54%)	95% CI: 49.3%–58.6%				191 (59.3%)	95% CI: 53.7%–64.7%			50 (39.4%)	95% CI: 30.8%–48.4%		
Neuropathic pain	Neuropathic Pain Questionnaire^b^ (NPQ) (*Neuropathic pain [> 0]* or *non‐neuropathic pain [< 0]*)	400	−0.38	1.08	−2.25–2.79	119 (22.9%)	277	−0.33	1.11	−2.25–2.79	118	−0.54	0.98	−1.76–2.65
	NP Prevalence (> 0)	120 (30%)	95% CI: 25.6%–34.8%				92 (33.2%)	95% CI: 27.7%–39.1%			24 (20.3%)	95% CI: 13.5%–28.7%		
Pain‐related worrying	Pain Catastrophisation Scale (PCS) (0–52)	474	15.94	14.69	0–52	45 (8.7%)	335	17.05	14.98	0–52	134	12.95	13.58	0–52
	PCS‐Rumination	489	5.11	5.10	0–16	30 (5.8%)	346	5.50	5.17	0–16	138	4.12	4.81	0–16
	PCS‐Magnification	490	3.17	3.49	0–13	29 (5.6%)	347	3.88	3.55	0–13	138	3.20	3.25	0–12
	PCS‐Helplessness	480	7.29	6.96	0–24	39 (7.5%)	338	7.85	7.11	0–24	137	5.82	6.39	0–24
	Prevalence of Pain‐Related Worrying (PCS ≥ 30)	99 (20.9%)	95% CI: 17.3%–24.8%				80 (23.9%)	95% CI: 19.4%—28.8%			17 (12.7%)	95% CI: 7.6%—19.5%		
Self‐efficacy	Pain Self‐Efficacy Scale (PSEQ) (0–60)	476	37.43	15.84	0–60	43 (8.3%)	335	36.66	15.69	0–60	137	39.42	16.08	1–60
Kinesiophobia	Tampa Scale of Kinesiophobia (TSK‐11) (11–48)	427	24.16	7.90	11–44	92 (17.7%)	298	24.21	7.69	9–44	126	23.93	8.46	9–44
	Minimal (≤ 22)	179 (41.9%)					122 (40.9%)				57 (45.2%)			
	Low (23–28)	121 (28.3%)					90 (30.2%)				29 (23%)			
	Moderate (29–35)	93 (21.8%)					65 (21.8%)				27 (21.4%)			
	High (≥ 36)	34 (8%)					21 (7%)				13 (10.3%)			
	Clinically Significant Kinesiophobia (TSK ≥ 25)	213 (49.9%)	95% CI: 45%–54.7%				148 (49.7%)	95% CI: 43.9%–55.5%			62 (49.2%)	95% CI: 40.2%–58.3%		

*Note:* Participants who reported their gender as ‘*other*’ (*n* = 3) or ‘preferred not to say’ (*n* = 2) were excluded from stratified analysis based on gender due to insufficient sample size for meaningful comparison. Confidence Intervals (95% CI) calculated (binomial) to 95% on 15.12.24: https://sample‐size.net/confidence‐interval‐proportion/.

Abbreviations: BMI, Body Mass Index (Kg/m^2^); n, number of respondents; PROMs, patient reported outcome measures; SD, standard deviation.

^a^ *n* = Valid Sample.

^b^ NPQ Discriminant Function Score.

### 4.1 Socio‐Demographics

Participants had a mean age of 46.6 ± 12.7 years (range: 18–76) and were primarily female (*n* = 367; 70.7%), white (*n* = 499; 96.1%), and non‐smokers (*n* = 453; 88%). Most participants were married or partnered (*n* = 327; 65.5%); achieved education up to third level (*n* = 360; 69.4%); and employed full‐time or part‐time (*n* = 314; 60.5%) with 18.5% (*n* = 96) unable to work due to disability or illness.

### 4.2 Obesity and Health

The mean BMI at baseline for the total cohort was 46.8 ± 9.1 kg/m^2^ (range: 26–105.5). Mean BMI for female participants was 46.6 ± 8 kg/m^2^ (range: 26–84.8) and 47.2 ± 11.3 kg/m^2^ (range: 30.2–105.5) for males. Results for the stratified BMI classes were as follows: Class I: mean 33.1 ± 1.6 kg/m^2^ (*n* = 27; range: 30.2–34.9); Class II mean 37.4 ± 1.4 kg/m^2^ (*n* = 71; range: 35–39.9); and Class III mean 49.4 ± 8.2 kg/m^2^ (*n* = 416; range: 40–105.5). Baseline data for the King's Obesity Staging Criteria (KOSC) and Edmonton Obesity Staging System (EOSS) have been included in Tables 1A and 1C.

**TABLE 1C:** Baseline results for all sociodemographic and health‐related PROMs, stratified by BMI Class.

		Class I (BMI 30–34.9 kg/m^2^)				Class II (BMI 35–39.9 kg/m^2^)				Class III (BMI ≥ 40 kg/m^2^)			
		*n* =^a^	Mean	SD	Range	*n* =	Mean	SD	Range	*n* =	Mean	SD	Range
Sociodemographic													
Age		27	47.89	11.96	22–67	71	46.94	13.431	18–75	416	46.51	12.64	19–76
Gender	Total	27				71				416			
	Female	18 (66.7%)				42 (59.2%)				303 (72.8%)			
	Male	9 (33.3%)				27 (38%)				110 (26.4%)			
	Prefer not to say	0				0				2 (0.5%)			
	Other	0				2 (2.8%)				1 (0.2%)			
Ethnic background	Total	27				71				416			
	White	24 (88.9%)				66 (93%)				404 (97.1%)			
	Black	1 (3.7%)				0				4 (1%)			
	Asian	1 (3.7%)				3 (4.2%)				4 (1%)			
	Other	1 (3.7%)				2 (2.8%)				4 (1%)			
Relationship status	Total	25				71				398			
	Single	7 (258%)				24 (33.8%)				94 (23.6%)			
	Married	13 (52%)				34 (47.9%)				226 (56.8%)			
	Partnered	2 (8%)				5 (7%)				43 (10.8%)			
	Separated	0				6 (8.5%)				10 (2.5%)			
	Divorced	3 (12%)				0				5 (1.3%)			
	Widowed	0				2 (2.8%)				9 (2.3%)			
	Prefer not to say	0				0				11 (2.8%)			
Employment status	Total	27				71				416			
	Working Full‐time	11 (40.7%)				39 (54.9%)				200 (48.1%)			
	Working Part‐time	3 (11.1%)				8 (11.3%)				49 (11.8%)			
	Retired	2 (7.4%)				8 (11.3%)				34 (8.2%)			
	Unable to work due to disability	5 (18.5%)				10 (14.1%)				80 (19.2%)			
	Homemaker	4 (14.8%)				3 (4.2%)				25 (6%)			
	Student	0				1 (1.4%)				8 (1.9%)			
	Unemployed	2 (7.4%)				2 (2.8%)				20 (4.8%)			
Education background	Total	27				71				415			
	No formal education	0				1 (1.4%)				3 (0.7%)			
	Primary education	0				3 (4.2%)				20 (4.8%)			
	Lower secondary education	4 (14.8%)				7 (9.9%)				42 (10.1%)			
	Higher secondary education	1 (3.7%)				9 (12.7%)				66 (15.9%)			
	Technical or vocational qualification	5 (18.5%)				6 (8.5%)				57 (13.7%)			
	Third level (non‐degree)	4 (14.8%)				11 (15.5%)				79 (19%)			
	Third level (degree)	9 (33.3%)				16 (22.5%)				90 (21.7%)			
	Postgraduate degree	4 (14.8%)				18 (25.4%)				58 (14%)			
Obesity and health‐related outcomes													
Smoking status	Total	27				71				412			
	Yes, daily	2 (7.4%)				8 (11.3%)				31 (7.5%)			
	Yes, at least once a week	0				0				6 (1.5%)			
	Yes, less often than once a week	0				2 (2.8%)				12 (2.9%)			
	No, not at all	25 (92.6%)				61 (85.9%)				363 (88.1%)			
BMI (kg/m^2^)	Total	27	33.12	1.59	30.2–34.9	71	37.43	1.38	35–39.9	416	49.39	8.21	40–105.5

King's Obesity Staging Criteria (KOSC)		*n* = 2				*n* = 8				*n* = 33			
Stage		0 Normal health	1 At risk	2 Established disease	3 Advanced disease	0 Normal health	1 At risk	2 Established disease	3 Advanced disease	0 Normal health	1 At risk	2 Established disease	3 Advanced disease
	Airway	0	1 (50%)	1 (50%)	0	1 (12.5%)	4 (50%)	3 (37.5%)	0	6 (18.2%)	17 (51.5%)	9 (27.3%)	1 (3%)
	BMI	0	1 (50%)	1 (50%)	0	0	0	7 (87.5%)	1 (12.5%)	1 (3%)	1 (3%)	28 (84.8%)	3 (9.1%)
	Cardiovascular	1 (50%)	0	1 (50%)	0	1 (12.5%)	4 (50%)	2 (25%)	1 (12.5%)	11 (33.3%)	15 (45.5%)	6 (18.2%)	1 (3%)
	Diabetes	1 (50%)	0	1 (50%)	0	4 (50%)	1 (12.5%)	3 (37.5%)	0	23 (69.7%)	3 (9.1%)	6 (18.2%)	1 (3%)
	Economic	2 (100%)	0	0	0	4 (50%)	2 (25%)	2 (25%)	0	17 (51.5%)	12 (36.4%)	4 (12.1%)	0
	Functional	0	2 (100%)	0	0	1 (12.5%)	6 (75%)	1 (12.5%)	0	2 (6.1%)	27 (81.8%)	4 (12.1%)	0
	Gonadal Axis	1 (50%)	0	1 (50%)	0	5 (62.5%)	2 (25%)	1 (12.5%)	0	22 (66.7%)	7 (21.2%)	4 (12.1%)	0
	Health Status (Perceived)	1 (50%)	1 (50%)	0	0	2 (25%)	5 (62.5%)	1 (12.5%)	0	2 (6.1%)	26 (78.8%)	5 (15.2%)	0
	Image	0	1 (50%)	1 (50%)	0	7 (87.5%)	0	0	1 (12.5%)	5 (15.2%)	23 (69.7%)	5 (12.2%)	0
	Junction of gastro‐oesophagus	2 (100%)	0	0	0	3 (37.5%)	3 (37.5%)	2 (25%)	0	18 (54.5%)	9 (27.3%)	6 (18.2%)	0
	Kidney	0	0	2 (100%)	0	5 (62.5%)	1 (12.5%)	2 (25%)	0	23 (69.7%)	1 (3%)	9 (27.3%)	0
	Liver	0	1 (50%)	1 (50%)	0	6 (75%)	1 (12.5%)	1 (12.5%)	0	26 (78.8%)	4 (12.1%)	3 (9.1%)	0
	Medication	2 (100%)	0	0	0	5 (62.5%)	2 (25%)	1 (12.5%)	0	27 (81.8%)	6 (18.2%)	0	0
	Neurology	2 (100%)	0	0	0	8 (100%)	0	0	0	33 (100%)	0	0	0
	Other	2 (100%)	0	0	0	8 (100%)	0	0	0	32 (97%)	0	1 (3%)	0
Edmonton obesity staging system (EOSS)		2				3				74			
	Stage 1	0				0				0			
	Stage 2	1 (50%)				3 (100%)				23 (31.1%)			
	Stage 3	1 (50%)				0				48 (64.9%)			
	Stage 4	0				0				3 (4.1%)			
Comorbidities	Self‐Administered Comorbidity Questionnaire (SCQ) (0–36)	12	6	6	0–20	46	4.76	3.772	0–15	273	5.92	4.361	0–22
	Nil comorbidities	1 (8.3%)				6 (13%)				30 (11%)			
	1 comorbidity	5 (41.7%)				8 (17.4%)				49 (17.9%)			
	≥ 2 comorbidities	6 (50%)				32 (69.6%)				194 (71.1%)			
Health‐related	EuroQol‐ 5 Dimension (EQ‐5D‐5L): Utility Score (< 0 − +1)	25	0.72	0.31	−0.169–1	69	0.69	0.37	−0.580–1	399	0.54	0.38	−0.775–1
Quality of life	EQ‐5D‐5L: VAS Score (0–100)	24	60.83	22.49	20–100	69	62.55	20.25	5–98	402	52.48	19.70	0–100
Depression	Patient Health Questionnaire (PHQ9) (0–27)	22	7.59	4.87	0–17	66	8.38	7.00	0–27	387	10.34	6.95	0–27
	No/minimal Depression (0–4)	5 (22.7%)				24 (36.4%)				90 (23.3%)			
	Mild Depression (5–9)	10 (45.5%)				17 (25.8%)				103 (26.6%)			
	Moderate Depression (10–14)	5 (22.7%)				12 (18.2%)				90 (23.3%)			
	Moderately Severe Depression (15–19)	2 (9.1%)				9 (13.6%)				54 (14%)			
	Severe Depression (20–27)	0				4 (6.1%)				50 (12.9%)			
Mental well‐being	Warwick‐Edinburgh Mental Well‐Being Scale (WEMWBS) (14–70)	27	46.32	8.89	24–63	67	46.3	11.39	19–70	386	44.12	11.75	14–70

*Note:* Participants with a BMI of < 30 kg/m^2^ (*n* = 2) were excluded from analysis stratified by BMI due to insufficient sample size for meaningful comparison. Confidence Intervals (95% CI) calculated (binomial) to 95% on 15.12.24: https://sample‐size.net/confidence‐interval‐proportion/.

Abbreviations: BMI, Body Mass Index (Kg/m^2^); *n*, number of respondents; PROMs, patient reported outcome measures; SD, standard deviation.

^a^ *n* = Valid Sample.

### 4.3 Comorbidities

The SCQ has a score range of 0–36 with higher scores indicating greater and more adversely impactful comorbidities (Sangha et al. 2003). Based on a valid sample of *n* = 333, participants reported having none (*n* = 37; 11.1%); one (*n* = 62; 18.6%); or ≥ 2 comorbidities (*n* = 234; 70.3%) (SCQ: mean 5.82 ± 4.4; range: 0–22). Female participants reported a mean of 5.8 ± 4.3 (range: 0–22) and males a mean of 5.9 ± 4.7 (range: 0–20). Presence of comorbidities for the stratified BMI classes were as follows: Class I: mean 6 ± 6; range: 0–20; Class II: mean 4.8 ± 3.8 (range: 0–15); and Class III: mean 5.9 ± 4.4 (range: 0–22).

### 4.4 Health‐Related Quality of Life

The mean overall health‐related quality of life (0–100 EQ‐5D‐5L Visual Analog Scale [VAS]) was 54.2 ± 20.3 (range: 0–100), with higher scores indicating greater quality of life (Feng et al. 2021). The proportion of participants reporting moderate, severe or extreme health related quality of life problems were: mobility: 40.3% (95% CI: 36.7%–45.5%); self‐care: 14.2% (95% CI: 11.3%–17.6%); usual activities: 34.4% (95% CI: 30.2%–38.8%); pain/discomfort: 50.7% (95% CI: 46.8%–55.8%); anxiety/depression: 34.4% (95% CI: 30.2%–38.8%) (Supporting Information S3). For female participants, the mean EQ‐5D‐5L VAS score was 54.2 ± 20.7 (range: 0–100), while the male mean was 54.2 ± 19.6 (range: 0–100). Means for participants stratified according to BMI class were as follows: Class I: mean 60.8 ± 22.5 (range: 20–100); Class II: mean 62.6 ± 20.3; (range: 5–98); and Class III: mean 52.5 ± 19.7 (range: 0–100).

### 4.5 Depression and Mental Well‐Being

Mean depression for the total cohort was 10 ± 7 (range: 0–27) (PHQ9), range of 0–27 with higher scores indicating more severe depression (Cameron et al. 2008). Results stratified by depression severity for the total cohort at baseline were as follows: no/minimal depression: 25%; mild depression: 27.3%; moderate depression: 22.3%; moderately severe depression: 13.5%; and severe depression: 11.9%. Female mean depression was 10.5 ± 7.1 (range: 0–27), while males reported a lower mean of 8.7 ± 6.6 (range: 0–27). Mean results for the stratified BMI classes were comparable between Classes I and II, with Class III reporting slightly worse scores. Results were as follows: Class I: mean 7.6 ± 4.9 (range: 0–17); Class II: mean 8.4 ± 7 (range: 0–27); and Class III: mean 10.3 ± 7 (range: 0–27).

Mental well‐being was assessed using the WEMWBS (range 14–70, with lower scores indicating worse mental health) (Teague et al. 2018). The mean WEMWBS score for all participants was 44.5 ± 11.6 (range: 14–70), with females reporting slightly poorer mean mental health scores (43.9 ± 11.8; range: 14–70; versus males 46.2 ± 11.3; range: 14–70). Mental well‐being results for the stratified BMI classes were similar to the PHQ9 results, with Class III reporting slightly worse mean scores than Classes I and II: Class I: mean 46.3 ± 8.9 (range: 24–63); Class II: mean 46.3 ± 11.4 (range: 19–70); and Class III: mean 44.1 ± 11.8 (range: 14–70).

### 4.6 Pain Characteristics

#### 4.6.1 Pain Prevalence

Participants' pain point prevalence at baseline attending obesity treatment services was 77% (95% CI: 73.1%–80.6%).

#### 4.6.2 Pain Intensity

Mean pain intensity (0–10 NRS) was 3.9 ± 0.1 (range: 0–10), with higher scores reflecting more severe pain intensity (Jensen and Karoly 2011). Results stratified by pain intensity were as follows: no pain (NRS 0/10) 20.6% (*n* = 105); mild pain (1–4/10) 33.5% (*n* = 171); moderate pain (5–6/10) 23.9% (*n* = 122); and severe pain (≥ 7/10) 22% (*n* = 112).

#### 4.6.3 Pain Location

‘*Lower back*’ was the most frequently reported body pain site (55.1%; *n* = 286) followed by knees (‘*left knee’* 36.6%, *n* = 190; ‘*right knee*’ 40.3%, *n* = 209), hips (‘*left hip*’ 25.1%, *n* = 130; ‘*right hip*’ 12.3%, *n* = 134), ankle/ft (‘left ankle/foot’ 23.7%, *n* = 123; ‘right ankle/foot’ 25.1%, *n* = 130), and shoulders (‘*left shoulder*’ 20.8%, *n* = 108; ‘*right shoulder*’ 19.1%, *n* = 99) (Supporting Information S4). Participants reporting one pain site represented 6.6% (*n* = 34) of the cohort, while 9.4% (*n* = 47) reported two sites, and 61.4% (*n* = 318; 95% CI: 57.1%–65.6%) reported multisite pain (≥ 3 pain sites).

#### 4.6.4 Pain‐Related Disability

Lower back, upper limb, and lower limb pain‐related functional disability was measured using the RMDQ (range: 0–24, higher = worse, mean 5.6 ± 6.3), UEFI‐15 (range: 0–100, higher = worse, mean 79.1 ± 23.8) and LEFS (range: 0–100, lower = worse, mean 47.45 ± 21.8).

#### 4.6.5 Social Impact of Pain

The social impact of pain was measured using the PDI, with a scale range of 0–70 and higher scores indicating worse social impact (Tait et al. 1990). The mean PDI score was 24.8 ± 19.6 (range: 0–70).

#### 4.6.6 Central Sensitisation

The point prevalence of an assumed dominance of nociplastic pain was estimated using the CSI, with a cut‐off score ≥ 40 (scale range: 0–100), with higher scores indicating greater nociplastic symptoms (Neblett et al. 2013). Baseline point prevalence of nociplastic pain was 54% (*n* = 245; 95% CI: 49.3%–58.6%), with 34.1% (*n* = 155; 95% CI: 29.8%–38.7%) of participants reporting severe (50–59 CSI score) or extreme (≥ 60 CSI score) nociplastic pain symptoms.

#### 4.6.7 Neuropathic Pain

Neuropathic pain was assessed using the NPQ. Scores from the NPQ generated a discriminant function score with scores above and below zero, suggesting neuropathic pain (> 0) or non‐neuropathic pain (< 0) (Krause and Backonja 2003). Neuropathic pain was present in 30% of the cohort (*n* = 120; 95% CI: 25.6% to 34.8%; mean: −0.38 ± 1.08 range: −2.25 to 2.79).

#### 4.6.8 Pain‐Related Worrying

The point prevalence of clinically significant pain‐related worrying (PCS ≥ 30) was 20.9% (*n* = 99; 95% CI: 17.3%–24.8%) for the total cohort, with a complete scale range of 0–52 and greater scores representing greater levels of pain‐related worrying (Sullivan et al. 1995). Mean pain rumination was 5.1 ± 5.1 (range 0–16), while pain magnification was 3.2 ± 3.5 (range 0–13), and pain helplessness 7.3 ± 7 (range 0–24).

#### 4.6.9 Pain Self‐Efficacy

Self‐efficacy was assessed using the PSEQ with a scale range of 0–60 and higher scores indicating greater levels of confidence in dealing with pain (Nicholas 2007). Mean self‐efficacy was 37.4 ± 15.8 (range: 0–60).

#### 4.6.10 Kinesiophobia

Kinesiophobia was assessed using the TSK‐11, with a scale range of 11–48 and greater scores representing greater kinesiophobia (Woby et al. 2005). The point prevalence of clinically significant kinesiophobia (TSK ≥ 25) was 49.9% (*n* = 213; 95% CI: 45%–54.7%) with a mean score of 24.2 ± 7.9 (range: 9–44).

#### 4.6.11 Pain Treatments

Participants reported using a range and combination of pharmacological and non‐pharmacological treatments for pain; 37.4% (*n* = 194; CI: 33.2%–41.7%) reported using over the counter (OTC) medications (e.g., paracetamol, NSAIDs, or non‐opioid compound medications). Prescription medications (such as opioids or analgesia for ‘*nerve pain*’ or ‘*chronic pain*’) were used by 22.5% of participants (*n* = 116; CI: 19%–26.4%); while 30.3% (*n* = 157; CI: 26.3%–34.4%) of participants reported the use of alternative treatments (e.g., hot packs (6.4%; *n* = 33)), transcutaneous electrical nerve stimulation (TENS) (15%; *n* = 78), massage (4.6%; *n* = 24), mindfulness/mediation (9.8%; *n* = 51), and alternate therapies such as reiki, acupuncture (6.2%; *n* = 32). Pain treatment results have been included as Supporting Information S5.

### 4.7 Pain and Gender

No difference in prevalence was found between males and females (chi squared *p* = 0.068). While we found some statistically significant differences (Mean Difference (MD) females—males) in PROMs indicating worse pain in females compared to males, including pain intensity; number of pain locations; back, upper and lower limb pain‐related disability; central sensitisation; pain‐related worrying; and depression, none were judged as clinically significant (Table 2).

Furthermore, there were no statistically or clinically significant differences between females and males in age, BMI, levels of lower back pain‐related disability, social impact of pain, neuropathic pain, self‐efficacy, quality of life, or kinesiophobia.

Participants who indicated their gender as ‘*other*’ (*n* = 3) or ‘preferred not to say’ (*n* = 2) were excluded from stratified analysis based on gender due to insufficient sample sizes for meaningful comparison.

### 4.8 Pain and BMI

Stratified by BMI there were no statistically significant differences in pain prevalence across two of the three groups (Tables 1C and 1D). A statistical difference was found when comparing Class II to Class III, with a higher prevalence of pain found in BMI Class III (chi squared *p* = < 0.001) (Table 3).

**TABLE 1D:** Baseline results for all pain‐related PROMs, stratified by BMI class.

		Class I (BMI 30–34.9 kg/m^2^)				Class II (BMI 35–39.9 kg/m^2^)				Class III (BMI ≥ 40 kg/m^2^)			
		*n* =	Mean	SD	Range	*n* =	Mean	SD	Range	*n* =	Mean	SD	Range
Pain outcomes and PROMs													
Pain presence	Total	26				70				407			
	I have no pain	9 (34.6%)				28 (40%)				79 (19.4%)			
	I have pain, but am receiving treatment	10 (38.5%)				18 (25.7%)				133 (32.7%)			
	I have pain, but am not receiving treatment	7 (26.9%)				24 (34.3%)				195 (47.9%)			
	Pain Prevalence	17 (65.4%)		95% CI: 44.3%–82.8%		42 (60%)		95% CI: 47.6%–71.5%		328 (80.6%)		95% CI: 76.4%–84.3%	
Pain intensity	Numeric Rating Scale (NRS) (0–10)	25	3.64	3.32	0–10	68	2.68	2.87	0–10	412	4.18	2.82	0–10
	No pain	8 (32%)				26 (38.2%)				70 (17%)			
	Mild pain (1–4)	7 (28%)				25 (36.8%)				138 (33.5%)			
	Moderate pain (5–6)	5 (20%)				10 (14.7%)				107 (26%)			
	Severe pain (≥ 7)	5 (20%)				7 (10.3%)				97 (23.5%)			
Pain location	Michigan Body Map (MBM) (0–35)	27	4.48	4.43	0–13	70	4.11	6.28	0–35	416	5.19	5.14	0–26
	No pain sites	8 (29.6%)				25 (35.7%)				83 (20%)			
	1 Pain Site	1 (3.7%)				5 (7.1%)				28 (6.7%)			
	2 Pain Sites	3 (11.1%)				8 (11.4%)				37 (8.9%)			
	Prevalence Multisite pain (≥ 3 sites)	15 (55.6%)		95% CI: 35.3%–74.5%		32 (45.7%)		95% CI: 33.7%–58.1%		268 (64.4%)		95% CI: 59.6%—69%	
Pain‐related disability	Upper Extremity Functional Index UEFI‐15 (Interval score: 0–100)	22	76.89	28.47	0–100	65	85.58	22.5	3–100	367	79.05	23.39	0–100
	Lower Extremity Functional Scale (LEFS) (0–80)	24	54.79	25.28	4–80	66	57.88	19.08	5–80	380	45.24	21.31	0–80
	Roland Morris Disability Questionnaire (RMDQ) (0–24)	25	4.80	6.26	0–19	70	2.77	4.59	0–18	410	6.06	6.43	0–24
Social impact of pain	Pain Disability Index (PDI) (0–70)	21	18.48	19.14	0–60	65	15.85	16.64	0–64	372	26.61	19.52	0–70
Central sensitisation	Central Sensitisation Inventory (CSI) (0–100)	22	35.64	18.85	0–66	63	38.87	17.62	1–100	365	42.94	17.30	0–85
	Subclinical (0–29)	8 (36.4%)				19 (30.2%)				75 (20.5%)			
	Mild (30–39)	6 (27.3%)				18 (28.6%)				82 (22.5%)			
	Moderate (40–49)	2 (9.1%)				11 (17.5%)				76 (20.8%)			
	Severe (50–59)	3 (13.6%)				9 (14.3%)				67 (18.4%)			
	Extreme (60–100)	3 (13.6%)				6 (9.5%)				65 (17.8%)			
	Prevalence (CSI ≥ 40)	8 (36.4%)		95% CI: 17.2%–59.3%		26 (41.3%)		95% CI: 29%–54.4%		208 (57%)		95% CI: 51.7%–62.1%	
Neuropathic pain	Neuropathic Pain Questionnaire^b^ (NPQ) (*Neuropathic pain [> 0]* or *non‐neuropathic pain [< 0]*)	22	−0.17	1.13	−1.41–2.35	54	−0.65	1.01	−1.75–2.79	320	−0.36	1.08	−2.25–2.65
	NP Prevalence (> 0)	9 (40.9%)		95% CI: 20.7%–63.7%		10 (18.5%)		95% CI: 9.3%–31.4%		98 (30.6%)		95% CI: 25.6%–36%	
Pain‐related worrying	Pain Catastrophisation Scale (PCS) (0–52)	24	12.71	13.40	0–44	64	11.33	12.94	0–51	381	16.78	14.75	0–52
	PCS‐Rumination	25	4	4.74	0–14	65	3.91	4.69	0–16	394	5.35	5.12	0–16
	PCS‐Magnification	24	2.21	2.47	0–7	65	2.65	3.34	0–12	396	3.96	3.48	0–13
	PCS‐Helplessness	25	6.2	6.60	0–23	64	4.67	5.74	0–24	386	7.72	7.01	0–24
	Prevalence Pain‐Related Worrying (PCS ≥ 30)	3 (12.5%)		95% CI: 2.7%–32.4%		7 (10.9%)		95% CI: 4.5%—21.3%		87 (22.8%)		95% CI: 18.7%—27.4%	
Self‐efficacy	Pain Self‐Efficacy Scale (PSEQ) (0–60)	22	37.59	20.43	0–60	62	42.26	15.56	0–60	387	36.71	15.39	0–60
Kinesiophobia	Tampa Scale of Kinesiophobia (TSK‐11) (11–48)	21	22.14	8.63	11–37	54	22.63	8.27	9–44	347	24.43	7.643	11–43
	Minimal (≤ 22)	12 (57.1%)				25 (46.3%)				140 (40.3%)			
	Low (23–28)	3 (14.3%)				16 (29.6%)				102 (29.4%)			
	Moderate (29–35)	4 (19%)				9 (16.7%)				80 (23.1%)			
	High (≥ 36)	2 (9.5%)				4 (7.4%)				25 (7.2%)			
	Clinically Significant Kinesiophobia (TSK ≥ 25)	8 (38.1%)		95% CI: 18.1%–61.6%		24 (44.4%)		95% CI: 30.9%–58.6%		178 (51.3%)		95% CI: 45.9%–56.7%	

*Note:* Participants with a BMI of < 30 kg/m^2^ (*n* = 2) were excluded from analysis stratified by BMI due to insufficient sample size for meaningful comparison. Confidence Intervals (95% CI) calculated (binomial) to 95% on 15.12.24: https://sample‐size.net/confidence‐interval‐proportion/.

Abbreviations: BMI, Body Mass Index (Kg/m^2^); n, number of respondents; PROMs, patient reported outcome measures; SD, standard deviation.

^a^ *n* = Valid Sample.

^b^ NPQ Discriminant Function Score.

**TABLE 2:** Exploratory analysis of pain differences based on gender.

Domain (outcome measure range)		Valid sample and sample means					Mean difference (Females)—(Males)^a^, ^b^			
		*n* = (Valid sample)	Total sample mean	Female mean	Male mean	Two‐sided *p* value	Mean diff	Lower 95% CI	Upper 95% CI	Cohen's *d* point estimate
Age		519	46.610	46.420	47.730	0.287	−1.308	−3.718	1.102	−0.104
BMI (kg/m^2^)		516	46.810	46.629	47.150	0.613	−0.520	−2.547	1.506	−0.057
Health‐related										
Health‐related quality of life	EQ‐5D‐5L: Utility Score (< 0 − +1)	498	0.568	0.553	0.612	0.106	−0.059	−0.131	0.013	−0.153
	EQ‐5D‐5L: VAS Score (0–100)	500	54.220	54.210	54.170	0.984	0.041	−3.914	3.995	0.002
Depression	PHQ9 (0–27)	480	10	10.480	8.690	0.011	1.787	0.416	3.158	0.258
Mental well‐being	WEMWBS (14–70)	480	44.470	43.890	46.150	0.056	−2.263	−4.587	0.061	−0.195
Pain										
Pain intensity	NRS (0–10)	510	3.970	4.170	3.430	0.011	0.734	0.168	1.299	0.253
Pain location	MBM (0–35)	518	5.060	5.540	3.530	< 0.001	2.008	1.186	2.829	0.391
Pain‐related disability	UEFI‐15 (0–100)	458	79.090	77.110	86.240	< 0.001	−9.124	−13.357	−4.891	−0.389
	LEFS (0–80)	475	47.440	46.130	50.900	0.033	−4.774	−9.150	−0.398	−0.220
	RMDQ (0–24)	518	5.560	5.730	4.740	0.109	0.985	−0.220	2.191	0.157
Social impact of pain	PDI (0–70)	463	24.830	24.840	24.670	0.932	0.172	−3.800	4.143	0.009
Central sensitisation	CSI (0–100)	454	42.080	44.110	36.120	< 0.001	7.997	4.460	11.534	0.466
Neuropathic pain	NPQ^c^	400	−0.380	−0.329	−0.536	0.066	0.207	−0.014	0.428	0.193
Pain‐related worrying	PCS (0–52)	474	15.940	17.050	12.950	0.006	4.106	1.175	7.037	0.281
Self‐efficacy	PSEQ (0–60)	476	37.430	36.660	39.420	0.085	−2.767	−5.916	0.383	−0.175
Kinesiophobia	TSK‐11 (11–48)	427	24.160	24.210	23.930	0.734	0.286	−1.369	1.942	0.036

^a^ Mean Diff: (Females)—(Males).

^b^ Participants who indicated their gender as ‘*other*’ (*n* = 3) or ‘preferred not to say’ (*n* = 2) were excluded from stratified analysis based on gender due to insufficient sample size for meaningful comparison.

^c^ NPQ Discriminant Function Score: Neuropathic pain [> 0] or non‐neuropathic pain [< 0].

**TABLE 3:** Exploratory analysis of pain differences based on BMI (kg/m^2^).

Domain (outcome measure range)								Mean differences (MD)^a^ ^,^ ^b^											
		Total Valid Sample *(n = 519)*	Total Sample Mean	Class 1 Mean *(n = 27)*	Class 2 Mean *(n = 71)*	Class 3 Mean *(n = 416)*	ANOVA Sig	(Class 1)—(Class 2)				(Class 1)—(Class 3)				(Class 2)—(Class 3)			
								Mean Diff	Lower 95% CI	Upper 95% CI	Cohen's *d*	Mean Diff	Lower 95% CI	Upper 95% CI	Cohen's *d*	Mean Diff	Lower 95% CI	Upper 95% CI	Cohen's *d*
Age		519	46.61	47.89	46.94	46.51	0.84	0.95	−4.91	6.80	0.07	1.38	−3.54	6.29	0.11	0.43	−2.79	3.65	0.03
Health‐related																			
Health‐related	EQ‐5D‐5L: Utility Score (< 0 − +1)	498	0.57	0.72	0.69	0.54	0.001	0.03	−0.13	0.20	0.10	0.18	0.03	0.34	0.48	0.15	0.05	0.25	0.39
Quality of life	EQ‐5D‐5L: VAS Score (0–100)	500	54.22	60.83	62.55	52.48	< 0.001	−1.72	−11.53	8.09	−0.08	8.36	0.15	16.56	0.42	10.08	5.01	15.14	0.51
Depression	PHQ9 (0–27)	480	10	7.59	8.38	10.34	0.03	−0.79	−3.50	1.92	−0.12	−2.75	−5.00	−0.49	−0.40	−1.96	−3.78	−0.14	−0.28
Mental well‐being	WEMWBS (14–70)	480	44.47	46.32	46.3	44.12	0.28	0.02	−5.27	5.31	0.002	2.19	−2.81	7.20	0.19	2.17	−0.87	5.22	0.19
Pain																			
Pain intensity	NRS (0–10)	510	3.97	3.64	2.68	4.18	< 0.001	0.96	−0.43	2.35	0.32	−0.54	−1.70	0.61	−0.19	−1.51	−2.24	−0.78	−0.53
Pain location	MBM (0–35)	518	5.06	4.48	4.11	5.19	0.25	0.37	−2.26	2.99	0.06	−0.71	−2.70	1.28	−0.14	−1.08	−2.43	0.27	−0.20
Pain‐related disability	UEFI‐15 (0–100)	458	79.09	76.89	85.58	79.05	0.10	−8.69	−20.52	3.13	−0.36	−2.16	−12.39	8.06	−0.09	6.53	0.38	12.68	0.28
	LEFS (0–80)	475	47.44	54.79	57.88	45.24	< 0.001	−3.09	−14.62	8.45	−0.15	9.55	0.63	18.47	0.44	12.64	7.50	17.78	0.60
	RMDQ (0–24)	518	5.56	4.44	2.77	5.97	< 0.001	1.67	−0.97	4.32	0.33	−1.52	−4.03	0.98	−0.24	−3.20	−4.45	−1.95	−0.52
Social impact of pain	PDI (0–70)	463	24.83	18.48	15.85	26.61	< 0.001	2.63	−5.99	11.25	0.15	−8.13	−16.73	0.47	−0.42	−10.76	−15.33	−6.20	−0.56
Central sensitisation	CSI (0–100)	454	42.08	35.64	38.87	42.94	0.05	−3.24	−12.07	5.60	−0.18	−7.30	−14.81	0.20	−0.42	−4.06	−8.72	0.59	−0.23
Neuropathic pain	NPQ^c^	400	−0.38	−0.17	−0.65	−0.36	0.11	0.48	−0.05	1.00	0.46	0.18	−0.29	0.65	0.17	−0.30	−0.61	0.01	−0.28
Pain‐related worrying	PCS (0–52)	474	15.94	12.71	11.33	16.78	0.01	1.38	−4.84	7.60	0.11	−4.07	−10.15	2.00	−0.28	−5.45	−9.31	−1.60	−0.38
Self‐efficacy	PSEQ (0–60)	476	37.43	37.59	42.26	36.71	0.04	−4.67	−13.03	3.70	−0.28	0.88	−8.29	10.05	0.06	5.55	1.40	9.69	0.36
Kinesiophobia	TSK‐11 (11–48)	427	24.16	22.14	22.63	24.43	0.15	−0.49	−4.78	3.80	−0.06	−2.28	−5.69	1.12	−0.30	−1.80	−4.02	0.43	−0.23

*Note:* Participants with a BMI of < 30 kg/m^2^ (*n* = 2) were excluded from analysis stratified by BMI due to insufficient sample size for meaningful comparison.

^a^ BMI, Body Mass Index [BMI 1 = BMI Class I; BMI 2 = BMI Class II; BMI 3 = BMI Class III].

^b^ For sample sizes per outcome measure and per BMI Class, please refer to Tables 1C and 1D.

^c^ NPQ Discriminant Function Score: Neuropathic pain [> 0] or non‐neuropathic pain [< 0].

Between‐group comparisons (independent sample *t*‐testing) revealed no statistically significant differences between BMI Class I versus Class II for any pain‐related PROMs. However, differences were noted between Class I versus Class III obesity for three PROMs, with Class III having worse scores on self‐reported (i) health‐related quality of life EQ‐5D‐5L Utility; health‐related quality of life EQ‐5D‐5L VAS score; (ii) depression PHQ9 and (iii) lower limb pain‐related disability LEFS. For all three outcome measures, the effect sizes were small to moderate, and only one was potentially clinically significant: lower limb pain‐related disability.

Differences were also noted between Class II versus Class III obesity in the following nine of 15 PROMs: health‐related quality of life EQ‐5D‐5L utility scores and health‐related quality of life EQ‐5D‐5L VAS scores; depression PHQ9; pain intensity NRS; upper limb pain‐related disability UEFI‐15; lower limb pain‐related disability LEFS; back pain‐related disability RMDQ; social impact of pain PDI; pain‐related worrying PCS; and pain self‐efficacy PSEQ.

In all nine PROMs where differences were found, participants with Class III obesity had worse scores in all PROMs than those with Class II obesity. However, of the nine scores, only four of these represented clinically significant differences in scores (MD (Class II—Class III) (95% CI); *d*: likely degree of clinical difference): health‐related quality of life EQ‐5D‐5L VAS score (MD: 10.076; *d*: 0.509; a likely moderate clinical difference); lower limb pain‐related disability LEFS (MD: 12.639; *d*: 0.602; likely moderate clinical difference); social impact of pain PDI (MD: −10.764; *d*: −0.563; likely moderate clinical difference); and pain‐related worrying PCS (MD: −5.454; *d*: −0.376; likely small‐to‐moderate clinical difference).

Comparison of means revealed there were no significant differences (ANOVA *p* ≥ 0.05) between the three groups for age, mental well‐being, pain location, upper limb pain‐related disability, central sensitisation, neuropathic pain and kinesiophobia.

## 5 Discussion

Our multicentre study presents a baseline multidimensional biopsychosocial pain profile for adults attending obesity treatment services in Ireland. These findings further highlight heterogeneity in the multidimensional pain experiences in PwO. The pain (point) prevalence estimate identified in our study (77%; 95% CI: 73.1%–80.6%) is at the upper end of previous estimates (17.9%–91%) (Zhong et al. 2024; MacLellan et al. 2017), suggesting that pain is common among PwO attending obesity treatment services (Stone and Broderick 2012; Yong et al. 2022).

To the best of our knowledge, the prevalence of nociplastic pain in PwO has not previously been reported. Nociplastic pain likely occurs in a non‐linear continuum involving upregulation of neuro‐immune pathways that result in persistent pain with peripheral and/or central sensitization (IASP 2020; Kosek 2024). It is characterised by widespread body pain, often co‐occurring with fatigue and altered sleep, cognition and mood, as well as multisensory hypersensitivity (Kaplan et al. 2024). Results from our study indicate a likely baseline prevalence of 54% for PwO with nociplastic pain. Prevalence estimates of nociplastic pain vary greatly across populations, although the prevalence of nociplastic pain in PwO appears comparable to those living with rheumatoid arthritis (RA) (Ohno et al. 2020) and osteoarthritis (OA) (Zheng and Chen 2015). Due to (i) overlap in populations; and (ii) parallel adiposity‐associated pro‐inflammatory mechanisms (Wang and He 2018). However prevalence estimates for pain may be heterogenous due to screening methods, sample selection, and bias—which could account for some variations in estimates across overlapping groups. The high prevalence of reported multisite pain further suggests nociplastic pain is an area of focus in PwO. The distribution of pain across the body may be more important than site‐specific location and pain treatment (Tanguay‐Sabourin et al. 2023). This also further supports the heterogeneity, complexity and need for multidimensional pain assessment in PwO.

Neuropathic pain was indicated in a third (30%) of participants in this study. A cross‐sectional study based on a UK Biobank cohort reported a 9.2% (*n* = 13,734) prevalence of neuropathic pain, consistent with estimates of 7%–10% prevalence in the general population (Baskozos et al. 2023). While there have been no previous estimates of neuropathic pain prevalence in PwO specifically, some studies have reported estimates of 20%–40.8% in people with diabetes, with higher BMI and obesity being independently associated with increased neuropathic pain (Baskozos et al. 2023; Callaghan et al. 2020). Other risk factors and associations for neuropathic pain have been identified as female gender and being from an area of social deprivation, higher BMI, with some conflicting evidence regarding age (Smith et al. 2020).

Baseline estimates of nociplastic pain could be useful for clinicians for differentiating and focusing treatments acutely, but also for prognostically recognising nociplastic pain as a precursor to the evolution to chronicity (Ferro Moura Franco et al. 2021; Klyne et al. 2019). Factors for the transition of nociceptive to chronic pain involve negative affective features, such as low mood (Tanguay‐Sabourin et al. 2023), which amplify and prolong pain perception via maladaptive thought processes such as pain‐related worrying, catastrophisation, or excessive fear‐association with movement (kinesiophobia) (Cohen et al. 2021; Kaplan et al. 2024). Kinesiophobia and pain‐related worrying have previously been associated as prognostic factors for increased pain‐related disability in PwO (Smith et al. 2020; Varallo et al. 2020) as previously described under the Fear Avoidance Model (FAM) (Crombez et al. 2012). Results in this study highlighting high levels of kinesiophobia and pain‐related fear in PwO may be used to help clinicians to identify barriers to treatment and better stratify PwO most at risk for pain‐related disability. Furthermore, kinesiophobia and pain‐related fear can be targeted to improve outcomes for treatment strategies that include exercise‐based interventions for PwO and pain.

Using mechanism‐based classification of pain could lead to more effective pain management for PwO. This includes more timely and tailored pain management programmes (PMPs) for PwO, strategies targeted towards mechanisms‐based classifications of pain and the avoidance of ineffective and potentially harmful treatments that could risk pain chronification (Klyne et al. 2019). Treatment of nociplastic pain could invite clinicians to adopt a multimodal approach. Non‐pharmacological therapies are the first‐line treatments, including pain science education, cognitive behavioural therapy (CBT), acceptance and commitment therapy, exercise, and goal‐oriented pain management that can include pacing strategies (Ferro Moura Franco et al. 2021). Effective pharmacotherapy for nociplastic pain is challenging and traditional analgesia (paracetamol, NSAIDs) is likely to be ineffective (Kosek 2024). The use of opioids has been discouraged due to the potential worsening of hyperalgesia and interference with sleep (Fitzcharles et al. 2021), both of which are commonly observed in PwO (Chin et al. 2020). Despite this, obesity remains associated with more long‐term opioid use compared to those without obesity (Stokes et al. 2019). Recognising the potential mechanistic heterogeneity of pain in PwO could invite a shift towards mechanism‐informed multimodal treatment.

In contrast with the literature, our study found no clinically significant differences in any PROM by gender. Existing literature suggests that females typically experience pain more often, more intense pain, and greater levels of pain‐related depression than males (Keogh 2025; Le LH et al. 2024; Parisien et al. 2025). Some studies have previously reported stronger pain‐related worrying in women (Edwards et al. 2004). The evidence supporting gender‐associated differences suggests a higher risk of pain chronification in women compared to men, based on biological, psychological, and social factors (Keogh 2025; Parisien et al. 2025). Presently, the relationship between pain, gender, and obesity remains unclear. The reason for the absence of differences in pain between male and female PwO is not known. We wonder if aspects of the pathophysiology and/or experience of obesity may moderate the association between gender and multiple dimensions of the pain experience. Future research could prospectively explore this.

Exploratory analyses found when compared to PwO with a BMI < 40 kg/m^2^, PwO with a BMI ≥ 40 kg/m^2^ were found to have statistically significant worse health‐related quality of life, depression, pain intensity, upper and lower limb pain‐related disability, back pain, social impact of pain, pain‐related worrying, and pain self‐efficacy. These findings of increased pain associated with increased BMI are supported by previous findings (Garcia et al. 2024). We speculate whether these results are suggestive of a potential threshold effect where the multidimensional aspects of pain coalesce for those with a BMI ≥ 40 kg/m^2^, resulting in greater pain prevalence and pain‐related functional disability, with greater social impact of pain and worse health‐related quality of life. Furthermore, no significant differences in the prevalence of nociplastic or neuropathic pain were found across groups stratified by BMI. This highlights the complexity of mechanisms‐based classifications of pain and is suggestive of non‐BMI‐dependent drivers or moderators of pain in PwO (Deng et al. 2025).

These findings suggest that the clinical assessment and management of pain should be considered a core component of obesity management, rather than as a secondary consequence of excess adiposity. This merits more explicit recognition within obesity‐related clinical practice guidelines. Further exploration of the trajectories of the pain experiences in PwO in our cohort is planned, where potentially confounding sociodemographic (age, gender, ethnicity, relationship status, employment status and educational level) and clinical (comorbidities, depression) parameters will be adjusted for statistically by including these as covariates in the analysis.

This study should be considered within the following strengths and limitations. This was a multi‐centre study with participants from both publicly and privately funded clinics. To our knowledge, this is the first study to comprehensively investigate and report the multidimensional biopsychosocial nature of pain experienced by PwO attending obesity treatment services. Furthermore, this contributes to theme eight of the European Pain Federation's Pain Research Strategy, to *better understand and address comorbidities in pain* (Pickering et al. 2025). This study undertook a methodologically rigorous approach. The protocol was published open‐access, following a pre‐specified analysis plan, with any deviations from protocol being transparently reported.

Participants were English‐speaking adults, recruited from three obesity treatment services in Ireland. As such, the findings from this study may not be generalisable geographically, to different healthcare systems, or to PwO more generally, including those not engaged with healthcare services. Pain prevalence estimates are heterogenous due to several factors. Pain duration (acute versus chronic) was not assessed at baseline, limiting contextual interpretation of the reported pain profiles. BMI categories were used to operationally define subgroups for analysis. This is a potential limitation as BMI is a measure that fails to reflect metabolic health, nor the distribution nor dysfunction of adiposity (Frühbeck et al. 2019).

## 6 Conclusions

This study describes the multidimensionality and complexity of pain in PwO. The majority (77%) of PwO attending specialist obesity treatment services report experiencing pain. The intensity, nature and impact of their pain varies. Over half of the cohort reported pain that could be mechanistically classified as nociplastic, one third reported neuropathic pain, one fifth had clinically significant levels of pain‐related worrying and half reported kinesiophobia. By investigating the biopsychosocial multidimensional pain profiles of PwO, (e.g., mechanisms‐based classifications) our study adds to the understanding of their pain experience in a way that could usefully inform its assessment and treatment.

## Author Contributions

N.S.H. is the lead author and guarantor. K.M.S. planned the study, and K.M.S. and N.S.H. led the drafting and revising of the manuscript. N.S.H. was responsible for recruitment, data collection and the monitoring of study participants. J.O., C.G.D., C.W.L.R. and F.M.F. had responsibility for overseeing participant recruitment at each study site. N.S.H., K.M.S., and C.B. all contributed to the data analysis. N.S.H., K.M.S., C.G.D., C.D., C.B., B.M.F., J.O., C.W.L.R., F.M.F., G.O.D., S.B. and F.F. contributed to drafting of the manuscript and revisions. All authors agreed on the submitted version of the manuscript. The authors attest that there was no use of Large Language Models (LLM) or generative artificial intelligence (AI) technology in the generation of text, figures, or other informational content of this manuscript.

## Funding

This study is part of an investigator‐initiated project funded by an unrestricted University College Dublin Ad Astra studentship (Ref: 65820). The funders had no role in any aspect of the study.

## Ethics Statement

The study received approval from the Ethics and Medical Research Committee of St Vincent's University Hospital, Dublin, Ireland (Reference No.: RS21‐059). The University College Dublin Human Research Ethics Committee approved ethical review exemption status for this study after meeting criteria for a low‐risk study, on 18th February 2022 (Reference No.: LS‐E‐22‐41‐Hinwood‐Smart).

## Conflicts of Interest

F.M.F. has received payment from the University of Michigan for membership of a Data Safety Monitoring Board of the LEAP and LEGEND randomised controlled trials, and from the Danish Diabetes Academy for reviewing grant applications. He is an investigator (unpaid) on two Novo Nordisk randomised controlled trials, REDEFINE 2 and REDEFINE 3. He is funded by a CÚRAM/Science Foundation Ireland Project Grant (ref 13/RC/2073‐P2). C.W.L.R. and S.B. have received payments in various formats from several sources, including European Coalition for People Living with Obesity and Novo Nordisk Ireland. These, along with other sources, are listed in their respective Conflicts of Interest (CoI) declarations. N.S.H., C.G.D., C.D., C.B., B.M.F., G.O.D., J.O., F.F., and K.M.S. have no conflicts to declare.

## Supporting information


**Data S1:** STROBE checklist.
**Data S2:** Guidance for Reporting Involvement of Patients and the Public (GRIPP2) Reporting Checklist.
**Data S3:** Baseline EQ‐5D‐5L Stratified Results.
**Data S4:** Pain Sites Reported at Baseline (Michigan Body Map).
**Data S5:** Baseline Current Pain Treatment.

## Data Availability

Further supporting information S1 relating to this study can be found on the European Union‐funded repository, Zenodo (DOI: 10.5281/zenodo.7407171). An anonymised copy of the dataset from this study will be published on the same repository within 12 months of completing the longitudinal study.
