# Supplementary material for: Pain in People Living With Obesity: Baseline Multidimensional Profiles, Prevalence and Biopsychosocial Factors From a Cohort Study
Source: Eur J Pain. 2026 Jul 3;30(6):e70317. doi: 10.1002/ejp.70317 (PMC13330943; doi:10.1002/ejp.70317)
Supplement: Supplementary file 1 — Data S1: STROBE checklist. Data S2: Guidance for Reporting Involvement of Patients and the Public (GRIPP2) Reporting Checklist. Data S3: Baseline EQ‐5D‐5L Stratified Results. Data S4: Pain Sites Reported at Baseline (Michigan Body Map). Data S5: Baseline Current Pain Treatment. [file EJP-30-0-s001.docx]

**Title:** Pain in People Living with Obesity: Baseline Multidimensional Profiles, Prevalence, and Biopsychosocial Factors from a Cohort Study

**Authors:** Natasha S. Hinwood^1,2,3^, Colin G. Dunlevy^4^, Catherine Doody^1,2^, Catherine Blake^1,2^, Bróna M. Fullen^1,2^, Gráinne O’Donoghue^1^, Jean O’Connell^4,5^, Carel W. Le Roux^6^, Francis M. Finucane^7,8^, Susie Birney^9^, Fionnuala Fildes^10^, Keith M. Smart^1,2^.

**Corresponding author:** Natasha S. Hinwood

**Address:** UCD School of Public Health, Physiotherapy and Sport Science, University College Dublin, Dublin, Ireland

**Email:** [natasha.hinwood@ucdconnect.ie](mailto:natasha.hinwood@ucdconnect.ie)

**Phone:** +353 1 716 6511

**Full name, department, institution, city, and country of all co-authors.**

^1^UCD School of Public Health, Physiotherapy and Sport Science, University College Dublin, Dublin, Ireland

^2^UCD Centre for Translational Pain Research, University College Dublin, Dublin, Ireland

^3^Physiotherapy Department, Beacon Hospital, Dublin, Ireland

^4^Centre for Obesity Management (COM), St Columcille’s and St Vincent’s University Hospitals, Dublin, Ireland

^5^UCD School of Medicine, University College Dublin, Dublin, Ireland

^6^Diabetes Complications Research Centre, University College Dublin, Dublin, Ireland

^7^School of Medicine, College of Nursing and Health Sciences, University of Galway, Galway, Ireland

^8^Bariatric Medicine Service, Centre for Diabetes, Endocrinology and Metabolism, Galway University Hospitals, Galway, Ireland

^9^ Irish Coalition for People Living with Obesity (ICPO), Dublin, Ireland

^10^Independent Patient Insight Partner, St. Vincent’s Private Hospital, Dublin, Ireland

# Supplementary Information:

1. STROBE checklist (pages 2-4)
2. GRIPP2 checklist (page 5)
3. Baseline EQ-5D-5L Stratified Results (pages 6-7)
4. Pain Sites Reported at Baseline (Michigan Body Map) (page 8)
5. Baseline Current Pain Treatment (page 9)

# Supplementary Information 1: STROBE checklist:

| **Supplementary Information 1: STROBE Checklist**  STrengthening the Reporting of OBservational studies in Epidemiology Checklist of items that should be included in reports of cohort studies | | | | |
| --- | --- | --- | --- | --- |
|  | Item No | Recommendation | Included in Protocol? / Notes |  |
| **Title and abstract** | 1 | (*a*) Indicate the study’s design with a commonly used term in the title or the abstract | Yes – included in title, abstract and main paper (cohort study) |  |
|  |  | (*b*) Provide in the abstract an informative and balanced summary of what was done and what was found | Yes – page 3 of main manuscript, also provided separately in abstract section |  |
| Introduction | | |  |  |
| Background/rationale | 2 | Explain the scientific background and rationale for the investigation being reported | Yes – outlined in protocol (Smart et al 2022) and in introduction of manuscript (pages 3-4) |  |
| Objectives | 3 | State specific objectives, including any prespecified hypotheses | Yes – outlined in protocol (Smart et al 2022) and in introduction and aims sections of manuscript (pages 3-4) |  |
| Methods | | |  |  |
| Study design | 4 | Present key elements of study design early in the paper | Yes – outlined in protocol (Smart et al 2022) and methods section of manuscript (pages 4-5) |  |
| Setting | 5 | Describe the setting, locations, and relevant dates, including periods of recruitment, exposure, follow-up, and data collection | Yes – outlined in protocol (Smart et al 2022) and methods section of manuscript (pages 4-5) |  |
| Participants | 6 | (*a*) Give the eligibility criteria, and the sources and methods of selection of participants. Describe methods of follow-up | Yes – outlined in protocol (Smart et al 2022) and methods section of manuscript (pages 4-5) |  |
|  |  | (*b*) For matched studies, give matching criteria and number of exposed and unexposed | Not applicable |  |
| Variables | 7 | Clearly define all outcomes, exposures, predictors, potential confounders, and effect modifiers. Give diagnostic criteria, if applicable | Yes – outlined in protocol (Smart et al 2022) and methods section of manuscript (pages 4-5) |  |
| Data sources/ measurement | 8* | For each variable of interest, give sources of data and details of methods of assessment (measurement). Describe comparability of assessment methods if there is more than one group | Yes – outlined in protocol (Smart et al 2022) and methods section of manuscript (pages 4-5) |  |
| Bias | 9 | Describe any efforts to address potential sources of bias | Yes – outlined in protocol (Smart et al 2022) and on pages 5 and 13 of manuscript |  |
| Study size | 10 | Explain how the study size was arrived at | Yes – outlined in protocol (Smart et al 2022) |  |
| Quantitative variables | 11 | Explain how quantitative variables were handled in the analyses. If applicable, describe which groupings were chosen and why | Yes – statistical analysis plan was outlined in protocol (Smart et al 2022) and methods section of manuscript (5). Further details are given in Results section (pages 7-12). |  |
| Statistical methods | 12 | (*a*) Describe all statistical methods, including those used to control for confounding | Yes – statistical analysis plan was outlined in protocol (Smart et al 2022) and methods section of manuscript (5). Further details are given in Results section (pages 5-10). |  |
|  |  | (*b*) Describe any methods used to examine subgroups and interactions | Yes – statistical analysis outlined in methods section of manuscript (5). Further details are given in Results section (pages 5-10). |  |
|  |  | (*c*) Explain how missing data were addressed | Yes – statistical analysis outlined in methods section of manuscript (5). Further details are given in Results section (pages 5-10). |  |
|  |  | (*d*) If applicable, explain how loss to follow-up was addressed | Not applicable – this manuscript deals with baseline data only. |  |
|  |  | (*e*) Describe any sensitivity analyses | Not applicable |  |
| Results | | |  |  |
| Participants | 13* | (a) Report numbers of individuals at each stage of study—eg numbers potentially eligible, examined for eligibility, confirmed eligible, included in the study, completing follow-up, and analysed | Yes – reported in abstract and results section (pages 5-10). |  |
|  |  | (b) Give reasons for non-participation at each stage | Yes – outlined in results section and in figure 1 (page 6) |  |
|  |  | (c) Consider use of a flow diagram | Yes – page 6 |  |
| Descriptive data | 14* | (a) Give characteristics of study participants (eg demographic, clinical, social) and information on exposures and potential confounders | Yes – outlined in results section and Tables 1(A) and 1 (B) |  |
|  |  | (b) Indicate number of participants with missing data for each variable of interest | Yes – outlined in results section and Tables 1(A) and 1 (B) |  |
|  |  | (c) Summarise follow-up time (eg, average and total amount) | Not applicable for baseline data only |  |
| Outcome data | 15* | Report numbers of outcome events or summary measures over time | Yes – outlined in results section and Tables 1(A), 1 (B), 1 (C), 1(D) |  |
| Main results | 16 | (*a*) Give unadjusted estimates and, if applicable, confounder-adjusted estimates and their precision (eg, 95% confidence interval). Make clear which confounders were adjusted for and why they were included | Yes – outlined in results section and Tables 1(A), 1 (B), 1 (C), 1(D), and Tables 2 and 3 |  |
|  |  | (*b*) Report category boundaries when continuous variables were categorized | Yes – outlined in results section and Tables 1(A), 1 (B), 1 (C), 1(D) |  |
|  |  | (*c*) If relevant, consider translating estimates of relative risk into absolute risk for a meaningful time period | Not applicable |  |
| Other analyses | 17 | Report other analyses done—eg analyses of subgroups and interactions, and sensitivity analyses | Yes – outlined in results section and Tables 2 and 3 |  |
| Discussion | | |  |  |
| Key results | 18 | Summarise key results with reference to study objectives | Yes – outlined in discussion and conclusion sections (pages 10 and 14) |  |
| Limitations | 19 | Discuss limitations of the study, taking into account sources of potential bias or imprecision. Discuss both direction and magnitude of any potential bias | Yes – outlined in protocol (Smart et al 2022) and in manuscript (page 14) |  |
| Interpretation | 20 | Give a cautious overall interpretation of results considering objectives, limitations, multiplicity of analyses, results from similar studies, and other relevant evidence | Yes – outlined in discussion and conclusion sections (pages 10 - 14) |  |
| Generalisability | 21 | Discuss the generalisability (external validity) of the study results | Yes – outlined in discussion and conclusion sections (pages 10 - 14) |  |
| Other information | | |  |  |
| Funding | 22 | Give the source of funding and the role of the funders for the present study and, if applicable, for the original study on which the present article is based | Yes – outlined in funding statement (page 14) |  |

# 2. Supplementary Information 2: Guidance for Reporting Involvement of Patients and the Public (GRIPP2) Reporting Checklist

| Supporting Information 2**: GRIPP2 short form** | | |
| --- | --- | --- |
| **Section and Topic** | **Item** | **Reported on page no.** |
| 1: Aim | Report the aim of PPI in the study | Yes – outlined in published protocol (Smart et al 2022) |
| 2: Methods | Provide a clear description of the methods used for PPI in the study | Yes – outlined in published protocol (Smart et al 2022) |
| 3: Study results | Outcomes—Report the results of PPI in the study, including both positive and negative outcomes | Yes – outlined in acknowledgements and authorship contributions sections (page 14) |
| 4: Discussion and conclusions | Outcomes—Comment on the extent to which PPI influenced the study overall. Describe positive and negative effects | Yes – outlined in acknowledgements and authorship contributions sections (page 14) |
| 5: Reflections/critical perspective | Comment critically on the study, reflecting on the things that went well and those that did not, so others can learn from this experience | Yes – page 14 (study strengths and limitations). |

1. *PPI* patient and public involvement

# 3. Supplementary Information 3: Baseline EQ-5D-5L Stratified Results

| Supplementary Info. 3: Baseline EQ-5D-5L Stratified Results | | | | | | | | | | | | | |
| --- | --- | --- | --- | --- | --- | --- | --- | --- | --- | --- | --- | --- | --- |
|  | | Mobility | | | | | | Self-Care | | | | | |
|  |  | Total | Female | Male | Class I | Class II | Class III | Total | Female | Male | Class I | Class II | Class III |
| Level 1 (No Problems) | n | 158 | 110 | 47 | 13 | 36 | 107 | 319 | 232 | 84 | 19 | 50 | 247 |
|  | % | 31.6% | 31.3% | 32.6% | 25% | 52.2% | 26.7% | 63.8% | 65.7% | 58.7% | 76% | 72.5% | 61.6% |
| Level 2 (Slight Problems) | n | 137 | 99 | 37 | 5 | 18 | 112 | 110 | 75 | 35 | 5 | 14 | 90 |
|  | % | 27.4% | 28.1% | 25.7% | 20% | 26.1% | 27.9% | 22.0% | 21.2% | 24.5% | 20% | 20.3% | 22.4% |
| Level 3 (Moderate Problems) | n | 114 | 82 | 31 | 4 | 10 | 100 | 53 | 32 | 20 | 1 | 4 | 47 |
|  | % | 22.8% | 23.3% | 21.5% | 16% | 14.5% | 24.9% | 10.6% | 9.1% | 14.0% | 4% | 5.8% | 11.7% |
| Level 4 (Severe Problems) | n | 79 | 53 | 25 | 3 | 3 | 72 | 18 | 14 | 4 | 0 | 1 | 17 |
|  | % | 15.2% | 15.1% | 17.4% | 11.1% | 4.3% | 18% | 3.6% | 4.0% | 2.8% | 0% | 1.4% | 4.2% |
| Level 5 (Extreme Problems / Unable to Do) | n | 12 | 8 | 4 | 0 | 2 | 10 | 0 | 0 | 0 | 0 | 0 | 0 |
|  | % | 2.3% | 2.3% | 2.8% | 0% | 2.9% | 2.5% | 0% | 0% | 0% | 0% | 0% | 0% |
| **Total (n)** |  | **500** | **352** | **144** | **25** | **69** | **401** | **500** | **353** | **143** | **25** | **69** | **401** |
|  | | Usual Activities | | | | | | Anxiety / Depression | | | | | |
|  |  | Total | Female | Male | Class I | Class II | Class III | Total | Female | Male | Class I | Class II | Class III |
| Level 1 (No Problems) | n | 183 | 127 | 55 | 11 | 38 | 132 | 195 | 132 | 63 | 12 | 32 | 149 |
|  | % | 36.6% | 36% | 38.5% | 44% | 55.1% | 32.9% | 39.0% | 37.4% | 44.1% | 48% | 46.4% | 37.2% |
| Level 2 (Slight Problems) | n | 145 | 106 | 38 | 6 | 19 | 119 | 133 | 99 | 34 | 9 | 15 | 108 |
|  | % | 29% | 30% | 26.6% | 24% | 27.5% | 29.7% | 26.6% | 28% | 23.8% | 36% | 21.7% | 26.9% |
| Level 3 (Moderate Problems) | n | 112 | 74 | 36 | 3 | 9 | 99 | 111 | 75 | 33 | 4 | 15 | 92 |
|  | % | 22.4% | 21% | 25.2% | 12% | 13% | 24.7% | 22.2% | 21.2% | 23.1% | 16% | 21.7% | 22.9% |
| Level 4 (Severe Problems) | n | 47 | 34 | 13 | 4 | 3 | 39 | 34 | 25 | 8 | 0 | 3 | 31 |
|  | % | 9.4% | 9.6% | 9.1% | 16% | 4.3% | 9.7% | 6.8% | 7.1% | 5.6% | 0% | 4.2% | 7.7% |
| Level 5 (Extreme Problems / Unable to Do) | n | 13 | 12 | 1 | 1 | 0 | 12 | 27 | 22 | 5 | 0 | 4 | 21 |
|  | % | 2.6% | 3.4% | 0.7% | 4% | 0% | 3% | 5.4% | 6.2% | 3.5% | 0% | 5.8% | 5.2% |
| **Total (n)** |  | **500** | **353** | **143** | **25** | **69** | **401** | **500** | **353** | **143** | **25** | **69** | **401** |

| Supplementary Info. 4: Baseline EQ-5D-5L Stratified Results (continued) | | | | | | | |
| --- | --- | --- | --- | --- | --- | --- | --- |
|  | | Pain / Discomfort | | | | | |
|  |  | Total | Female | Male | Class I | Class II | Class III |
| Level 1 (No Problems) | n | 97 | 65 | 31 | 11 | 26 | 59 |
|  | % | 19.4% | 18.4% | 21.5% | 0.44 | 37.7% | 14.7% |
| Level 2 (Slight Problems) | n | 147 | 108 | 39 | 2 | 20 | 124 |
|  | % | 29.3% | 30.6% | 27.1% | 0.08 | 29.0% | 30.8% |
| Level 3 (Moderate Problems) | n | 150 | 96 | 53 | 9 | 15 | 126 |
|  | % | 29.9% | 27.2% | 36.8% | 0.36 | 21.7% | 31.3% |
| Level 4 (Severe Problems) | n | 81 | 64 | 15 | 1 | 5 | 74 |
|  | % | 15.6% | 18.1% | 10.4% | 0.04 | 7.2% | 18.4% |
| Level 5 (Extreme Problems / Unable to Do) | n | 26 | 20 | 6 | 2 | 3 | 19 |
|  | % | 5.2% | 5.7% | 4.2% | 0.08 | 4.3% | 4.7% |
| **Total (n)** |  | **501** | **353** | **144** | **25** | **69** | **402** |

# Supplementary Information 4: Pain Sites Reported at Baseline (Michigan Body Map)

| Supplementary Info 4: Pain Sites Reported at Baseline (Michigan Body Map) | | | | | | | | | | | | | | |
| --- | --- | --- | --- | --- | --- | --- | --- | --- | --- | --- | --- | --- | --- | --- |
| Body Location | Total | | Female | | Male | | | Class I | | | Class II | | Class III | |
|  | (n) | (%)¹ | (n) | (%)² | (n) | (%)³ | | (n) | (%)⁴ | (n) | | (%)⁵ | (n) | (%)⁶ |
| Left shoulder | 108 | 20.81% | 82 | 22.34% | 23 | 15.65% | | 7 | 25.93% | 9 | | 12.68% | 88 | 21.15% |
| Left upper arm | 37 | 7.13% | 30 | 8.17% | 6 | 4.08% | | 1 | 3.70% | 6 | | 8.45% | 30 | 7.21% |
| Left elbow | 32 | 6.17% | 24 | 6.54% | 6 | 4.08% | | 1 | 3.70% | 4 | | 5.63% | 26 | 6.25% |
| Left lower arm | 22 | 4.24% | 16 | 4.36% | 5 | 3.40% | | 1 | 3.70% | 3 | | 4.23% | 17 | 4.09% |
| Left wrist / hand | 74 | 14.26% | 59 | 16.08% | 13 | 8.84% | | 3 | 11.11% | 10 | | 14.08% | 59 | 14.18% |
| Left hip | 130 | 25.05% | 98 | 26.70% | 28 | 19.05% | | 3 | 11.11% | 12 | | 16.90% | 113 | 27.16% |
| Left groin | 34 | 6.55% | 28 | 7.63% | 5 | 3.40% | | 0 | 0% | 6 | | 8.45% | 27 | 6.49% |
| Left buttocks | 37 | 7.13% | 29 | 7.90% | 6 | 4.08% | | 2 | 7.41% | 6 | | 8.45% | 28 | 6.73% |
| Left upper leg | 54 | 10.40% | 44 | 11.99% | 9 | 6.12% | | 2 | 7.41% | 7 | | 9.86% | 44 | 10.58% |
| Left knee | 190 | 36.61% | 143 | 38.96% | 44 | 29.93% | | 8 | 29.63% | 19 | | 26.76% | 160 | 38.46% |
| Left lower leg | 67 | 12.91% | 50 | 13.62% | 15 | 10.20% | | 2 | 7.41% | 11 | | 15.49% | 53 | 12.74% |
| Left ankle / foot | 123 | 23.70% | 93 | 25.34% | 27 | 18.37% | | 9 | 33.33% | 14 | | 19.72% | 98 | 23.56% |
| Right shoulder | 99 | 19.08% | 81 | 22.07% | 16 | 10.88% | | 6 | 22.22% | 11 | | 15.49% | 80 | 19.23% |
| Right upper arm | 27 | 5.20% | 23 | 6.27% | 4 | 2.72% | | 3 | 11.11% | 4 | | 5.63% | 20 | 4.81% |
| Right elbow | 34 | 6.55% | 26 | 7.08% | 6 | 4.08% | | 3 | 11.11% | 5 | | 7.04% | 25 | 6.01% |
| Right lower arm | 17 | 3.28% | 14 | 3.81% | 2 | 1.36% | | 2 | 7.41% | 2 | | 2.82% | 12 | 2.88% |
| Right hip | 134 | 25.82% | 104 | 28.34% | 26 | 17.69% | | 5 | 18.52% | 13 | | 18.31% | 114 | 27.40% |
| Right wrist / hand | 64 | 12.33% | 51 | 13.90% | 10 | 6.80% | | 5 | 18.52% | 9 | | 12.68% | 48 | 11.54% |
| Right groin | 34 | 6.55% | 26 | 7.08% | 7 | 4.76% | | 0 | 0% | 5 | | 7.04% | 28 | 6.73% |
| Right buttock | 48 | 9.25% | 36 | 9.81% | 10 | 6.80% | | 3 | 11.11% | 4 | | 5.63% | 41 | 9.86% |
| Right upper leg | 66 | 12.72% | 50 | 13.62% | 15 | 10.20% | | 3 | 11.11% | 6 | | 8.45% | 57 | 13.70% |
| Right knee | 209 | 40.27% | 155 | 42.23% | 51 | 34.69% | | 8 | 29.63% | 28 | | 39.44% | 170 | 40.87% |
| Right lower leg | 66 | 12.72% | 46 | 12.53% | 18 | 12.24% | | 2 | 7.41% | 6 | | 8.45% | 57 | 13.70% |
| Right ankle / foot | 130 | 25.05% | 98 | 26.70% | 29 | 19.73% | | 5 | 18.52% | 18 | | 25.35% | 105 | 25.24% |
| Head | 58 | 11.18% | 49 | 13.35% | 6 | 4.08% | | 1 | 3.70% | 4 | | 5.63% | 52 | 12.50% |
| Face | 14 | 2.70% | 13 | 3.54% | 1 | 0.68% | | 0 | 0% | 2 | | 2.82% | 11 | 2.64% |
| Neck | 122 | 23.51% | 99 | 26.98% | 19 | 12.93% | | 8 | 29.63% | 10 | | 14.08% | 101 | 24.28% |
| Left Jaw | 23 | 4.43% | 18 | 4.90% | 3 | 2.04% | | 0 | 0% | 2 | | 2.82% | 20 | 4.81% |
| Right Jaw | 20 | 3.85% | 17 | 4.63% | 2 | 1.36% | | 0 | 0% | 3 | | 4.23% | 17 | 4.09% |
| Left chest / breast | 37 | 7.13% | 28 | 7.63% | 6 | 4.08% | | 2 | 7.41% | 2 | | 2.82% | 31 | 7.45% |
| Right chest / breast | 24 | 4.62% | 20 | 5.45% | 3 | 2.04% | | 2 | 7.41% | 2 | | 2.82% | 18 | 4.33% |
| Upper back | 88 | 16.96% | 73 | 19.89% | 12 | 8.16% | | 7 | 25.93% | 7 | | 9.86% | 72 | 17.31% |
| Lower back | 286 | 55.11% | 209 | 56.95% | 72 | 48.98% | | 12 | 44.44% | 29 | | 40.85% | 242 | 58.17% |
| Pelvis | 59 | 11.37% | 50 | 13.62% | 7 | 4.76% | | 2 | 7.41% | 8 | | 11.27% | 47 | 11.30% |
| Abdomen | 59 | 11.37% | 50 | 13.62% | 7 | 4.76% | | 3 | 11.11% | 6 | | 8.45% | 48 | 11.54% |
| No chronic pain | 99 | 19.08% | 62 | 16.89% | 37 | 25.17% | | 5 | 18.52% | 26 | | 36.62% | 68 | 16.35% |
| ¹Calculated from total participants (n=519) ²Calculated from total female participants (n=367) ³Calculated from total male participants (n=147) | | | | | | | ⁴Calculated from total participants in Class I Obesity (n=27) ⁵Calculated from total participants in Class II Obesity (n=71) ⁶Calculated from total participants in Class III Obesity (n=416) | | | | | | | |

# Supplementary Information 5: Baseline Current Pain Treatment

| Supplementary information 5: Baseline Current Pain Treatment | | | | | | | |
| --- | --- | --- | --- | --- | --- | --- | --- |
|  | | Total sample | Females | Male | Class I  (BMI 30 - 34.9 kg/m²) | Class II   (BMI 35 - 39.9 kg/m²) | Class III  (BMI ≥40 kg/m²) |
| Current pain treatment¹ n (%) | OTC Medication (e.g. paracetamol) | 20 (3.9%) | 17 (4.6%) | 2 (1.4%) | 1 (3.7%) | 1 (1.4%) | 17 (4.1%) |
|  | NSAIDs | 140 (27%) | 114 (31.1%) | 24 (16.3%) | 6 (22.2%) | 18 (25.4%) | 114 (27.4%) |
|  | Compound Medications | 99 (19.1%) | 81 (22.1%) | 18 (12.2%) | 2 (7.4% | 11 (15.5%) | 86 (20.7%) |
|  | Opioid Medications | 44 (8.5%) | 37 (10.1%) | 6 (4.1%) | 2 (7.4% | 3 (4.2%) | 38 (9.1%) |
|  | Neuropathic Medications | 51 (9.8%) | 40 (10.9%) | 10 (6.8%) | 1 (3.7%) | 4 (5.6%) | 45 (10.8%) |
|  | Medications for Chronic Pain | 39 (7.5%) | 33 (9%) | 6 (4.1%) | 3 (11.1%) | 4 (5.6%) | 31 (7.5%) |
|  | Hot Packs | 33 (6.4%) | 28 (7.6%) | 4 (2.7%) | 3 (11.1%) | 3 (4.2%) | 27 (6.5%) |
|  | TENS | 78 (15%) | 66 (18%) | 10 (6.8%) | 4 (14.8%) | 9 (12.7%) | 64 (15.4%) |
|  | Massage | 24 (4.6%) | 21 (5.7%) | 2 (1.4%) | 0 | 2 (2.8%) | 21 (5%) |
|  | Mindfulness | 51 (9.8%) | 42 (11.4%) | 8 (5.4%) | 1 (3.7%) | 7 (9.9%) | 43 (10.3%) |
|  | Alternate Therapies | 32 (6.2%) | 30 (8.2%) | 2 (1.4%) | 0 | 5 (7%) | 27 (16.6%) |
|  | Other² | 87 (16.8%) | 68 (18.5%) | 16 (10.9%) | 5 (18.5%) | 12 (16.9%) | 1. (16.6%) |
| 1. *Pain treatment options not mutually exclusive* 2. *Other' Treatments: physiotherapy (n=10); exercise / gym (n=5); ; swimming (n=5); ice baths / ice packs (n=5); baths / ‘hot tubs’ (n=3); steroid injections (n=4); attendance at pain management clinics or specialists (n=3); non-analgesic medications (n=3) (e.g. Valium, immunosuppressant medications); cognitive behavioural therapy (n=3); chiropractor treatments (n=2); CPAP therapy for sleeping (n=2); pressure / compression bandages (n=2); homeopathy / 'health products' (n=2); cannabis (n=1); aloe vera (n=1); and laser therapy (n=1).* 3. *Treatment categories were not mutually exclusive and were used for descriptive purposes only. Thus, there may be overlap between categories.* | | | | | | | |
